# Traceless Photopolymerization with Non‐Pulsed Red Light Enables 3D‐Printable Cell‐Laden Hydrogels

**DOI:** 10.1002/adma.202502386

**Published:** 2025-05-16

**Authors:** Ali Eftekhari, Kelsey Rianne de Graaf, Ekaterina Takmakova, Hatai Jongprasitkul, Alexander Efimov, Sanna Turunen, Andrew Kerr, Minna Kellomäki, Robert Luxenhofer, Timo Laaksonen, Nikita Durandin

**Affiliations:** ^1^ Faculty of Engineering and Natural Sciences Tampere University Tampere 33720 Finland; ^2^ Soft Matter Chemistry Department of Chemistry Faculty of Science University of Helsinki Helsinki PB55 Finland; ^3^ Biomaterials and Tissue Engineering Group Faculty of Medicine and Health Technology Tampere University Tampere 33720 Finland; ^4^ New Materials and Processes Group Faculty of Engineering Turku University of Applied Sciences Turku 20520 Finland; ^5^ Drug Research Program Faculty of Pharmacy University of Helsinki Helsinki 00014 Finland; ^6^ School of Natural and Environmental Sciences Newcastle University Newcastle‐upon‐Tyne NE1 7RU UK

**Keywords:** 3D printing, colorless, gelatine methacrylate (GelMA), hydrogels, methylene blue, photopolymerization, red light

## Abstract

Photocrosslinking of hydrogels with non‐pulsed red light offers improved biocompatibility and deep tissue penetration in contrast to traditional UV‐initiated methods. However, hydrogels fabricated upon red‐light excitation are always colored by a photoinitiator, limiting their use in applications requiring high optical transparency, such as (bio)sensors, ophthalmological applications, or wound dressings. Additionally, the cytotoxicity of a photoinitiator is always a concern, especially in bioapplications. Herein, a photoinitiating system composed of an FDA‐approved methylene blue photosensitizer and cytocompatible triethanolamine is introduced. The system can induce photopolymerization upon 625 nm irradiation and leaves no visible trace of the methylene blue color afterward, thus named “traceless”. With this approach, gelatine methacrylate hydrogel is successfully polymerized under ambient conditions. The hydrogel is permanently colorless with well‐controlled stiffness due to the light‐dependent nature of the polymerization process. The system is further successfully applied in extrusion‐based 3D‐bioprinting with NIH‐3T3 fibroblasts, followed by photocuring to produce cell‐laden 3D structures, indicating its potential for tissue engineering. Upon culturing the cell‐laden constructs, the fibroblasts are able to proliferate and adhere to the hydrogel material. The red‐light excitation enables polymerization through at least 5 mm of biological tissue, projecting, inter alia, its use for transdermal photopolymerization in minimally invasive implantation.

## Graphical abstract

The paper presents a red‐light‐induced polymerization process, initiated by the FDA‐approved dye methylene blue and the cytocompatible co‐initiator triethanolamine, for fabricating hydrogels. This photopolymerization process is oxygen‐tolerant, cytocompatible, and, most importantly, leaves no color traces, resulting in completely transparent scaffolds. The photoinitiating system is successfully applied for extrusion‐based 3D (bio)printing of cell‐laden hydrogels.

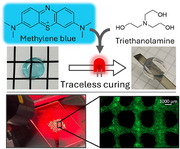

## 1 Introduction

Photopolymerization is initiated by reactions between molecules excited by exposure to light, as opposed to conventional, thermally initiated polymerization. Using light as the energy source to drive chemical reactions offers several unique advantages over conventional thermally induced polymerization: gentle reaction conditions, lower energy consumption, reduced environmental pollution, faster polymerization rates, as well as temporal and spatial control of the polymerization process.^[^ ^1^, ^2^, ^3^, ^4^, ^5^, ^6^, ^7^ ^]^ All these advantages support photopolymerization for the creation of bioscaffolds. In the past decades, photopolymerization has been predominantly focused on the UV range (<400 nm). However, UV light has numerous disadvantages, such as cellular damage upon absorption, ozone generation, low penetration depth, and unwanted side reactions, hindering their further applications in biological fields and fueling a desire to expand photopolymerization to longer wavelengths.^[^ ^8^, ^9^ ^]^

The development of visible light‐induced photopolymerization reactions, along with the advent of visible light‐emitting diodes (LEDs), addresses many of the previously mentioned issues.^[^ ^10^, ^11^ ^]^ Notably, within the visible range, red/near‐infrared (NIR) light possesses the deepest penetration depth in biological tissues, making it an exceptionally suitable candidate for applications in biomedicine.^[^ ^12^, ^13^, ^14^ ^]^ However, this naturally requires the utilization of dyes that absorb red/NIR light. These dyes, therefore, impart color to the materials, often resulting in polymers with inherent coloration.^[^ ^15^, ^16^ ^]^ In contrast, the creation of colorless polymers would yield significantly reduced optical density in the final product, which substantially aids in the homogeneous polymerization of thicker samples and improves the imaging of the materials.^[^ ^17^ ^]^

Photopolymerization reactions are categorized into two main types based on how they initiate radical formation. In type I reactions, photoinitiators directly generate radicals through high‐energy bond cleavage when exposed to light.^[^ ^18^ ^]^ This method is known for its rapid initiation but has limited applicability due to its reliance on high‐energy UV light. In contrast, Type II reactions function through either hydrogen atom abstraction, electron transfer, or energy transfer mechanisms, utilizing light wavelengths of ≥500 nm. This requires a multicomponent system comprising: i) a photocatalyst (PC) that acts as an initiator; ii) an electron‐rich donor (D) and/or an electron‐deficient acceptor (A) that serve as co‐initiators, working together to generate reactive radicals upon light irradiation.^[^ ^19^ ^]^ One well‐known example of a chromophore acting as a photocatalyst in type II photopolymerization reactions is methylene blue (MB^+^), a cationic dye that is FDA‐approved and known for its natural staining ability, as well as its medical applications, including treating methemoglobinemia and possessing anti‐malarial properties.^[^ ^20^ ^]^ MB^+^ has a maximum absorbance at 664 nm with a molar extinction coefficient of 9 × 10^4^ m ^−1^ cm ^−1^ and a 1 ns singlet state lifetime.^[^ ^21^ ^]^ Upon exposure to visible light, MB^+^ first gets excited to the singlet state and subsequently transforms into a triplet excited state (^3^MB^+^*) due to the efficient intersystem crossing (ISC). Its long triplet state lifetime of ≈32 µs makes MB^+^ a suitable photoinitiator for photoinduced electron transfer (PET) reactions in the presence of a suitable co‐initiator.^[^ ^22^ ^]^ To date, there have been some reports of PET polymerization utilizing MB^+^ as a photoinitiator (a photocatalyst). For instance, Matyjaszewski et al. utilized MB^+^ and X‐Cu/tris(2‐pyridylmethyl) amine (TPMA) as a pair of photoinitiator and electron donor, respectively, with Cu also acting as an oxygen scavenger.^[^ ^23^ ^]^ This approach was used to perform open‐to‐air photoinduced red‐light‐driven atom transfer radical polymerization (ATRP) in the mixture of deionized (DI) water and dimethyl sulfoxide (DMSO). In addition, Stansbury et al. utilized the pair MB^+^ and *N,N*‐diisopropylethylamine (DIPEA, aliphatic amine) in conjugation with diphenyl iodonium chloride (an oxidizing salt) to generate radicals in oxygen‐free conditions in a mixture of methanol, acetonitrile, and DI‐water.^[^ ^24^ ^]^ These recent studies utilizing MB^+^ as a photocatalyst are characterized by the distinct blue color of the final product, implying MB^+^ photocatalyst regeneration.^[^ ^24^ ^]^ In addition, some of these studies were conducted in non‐biocompatible environments and/or in the absence of oxygen.

Here, we report traceless, meaning producing colorless products, photopolymerization under ambient conditions in water and biological buffers, utilizing red light irradiation and MB^+^ as a sacrificial photosensitizer. Triethanolamine (TEA), used as a co‐initiator/reductant agent, reduces MB^+^ by PET and initiates radical polymerization. Performing this reaction in the presence of oxygen leads to the production of reactive oxygen species (ROS), which degrade MB^+^, resulting in a transparent, crosslinked hydrogel with no color traces in less than 10 minutes. The polymerization kinetics for the formation of a crosslinked hydrogel were investigated using in situ photorheology and ^1^H‐NMR. These techniques demonstrated the potential for reasonably fast polymer formation under low‐intensity red light irradiation. This photocuring system enables the fabrication of stable, colorless 3D structures under red light irradiation via extrusion‐based 3D printing. The system was further adapted for bioprinting NIH‐3T3 fibroblasts, yielding cell‐laden constructs with excellent adhesion and proliferation, highlighting its promise for tissue‐engineering applications.

## 2 Results and Discussion

**Figure 1:**
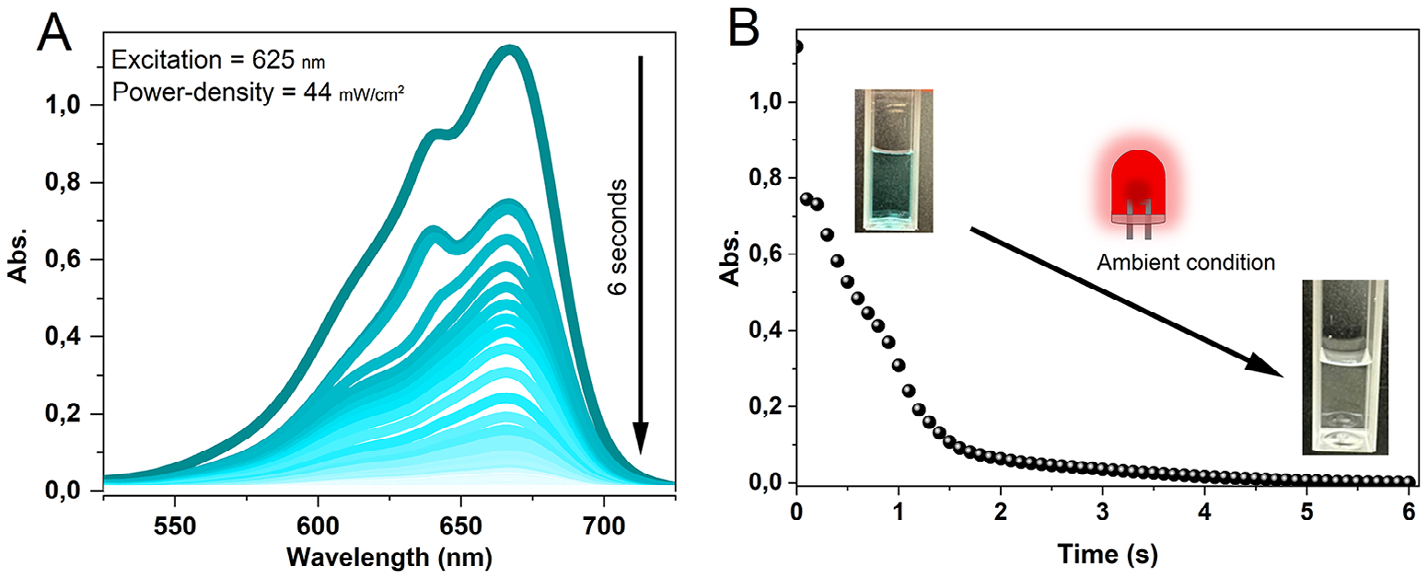
Absorption changes of MB^+^ (over time) in a solution consisting of 10% w/v GelMA, 37 mm TEA, and 4% v/v DMA, measured under ambient conditions. A) Absorption spectra of MB^+^ (one spectrum every 100 ms, total time 6 s) measured under continuous wave (cw) irradiation at 625 nm (44 mW cm^−2^). B) Absorbance monitored at the absorption maximum (664 nm).

Since combining photopolymerization and photobleaching of the photosensitizer is the main goal of this study, changes in absorption spectra of methylene blue upon irradiation were first investigated. The reduction of MB^+^ to Leuco‐MB in the presence of TEA under continuous‐wave (cw) irradiation at 625 nm (44 mW cm^−2^) resulted in a complete disappearance of the blue color (**Figure** **1**). This photoinduced process (Figure 1A,B) results in the formation of colorless leuco‐MB in less than 6 s under the present conditions, which include 16 µM MB^+^, 10 w/v % gelatine methacrylate (GelMA), 4 v/v% *N,N*‐dimethyl acrylamide (DMA), and 37 mm TEA in water under ambient conditions. The photoinduced process is power density‐dependent; it takes 23 s to completely transform MB^+^ when the power is reduced to 19 mW cm^−2^ (Figure S1, Supporting Information). Upon 625 nm irradiation, MB^+^ is first excited to its singlet state. Owing to a highly efficient ISC, it then transitions to the triplet excited state ^3^MB^+*^, as follows:
(1)

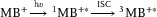




**Figure 2:**
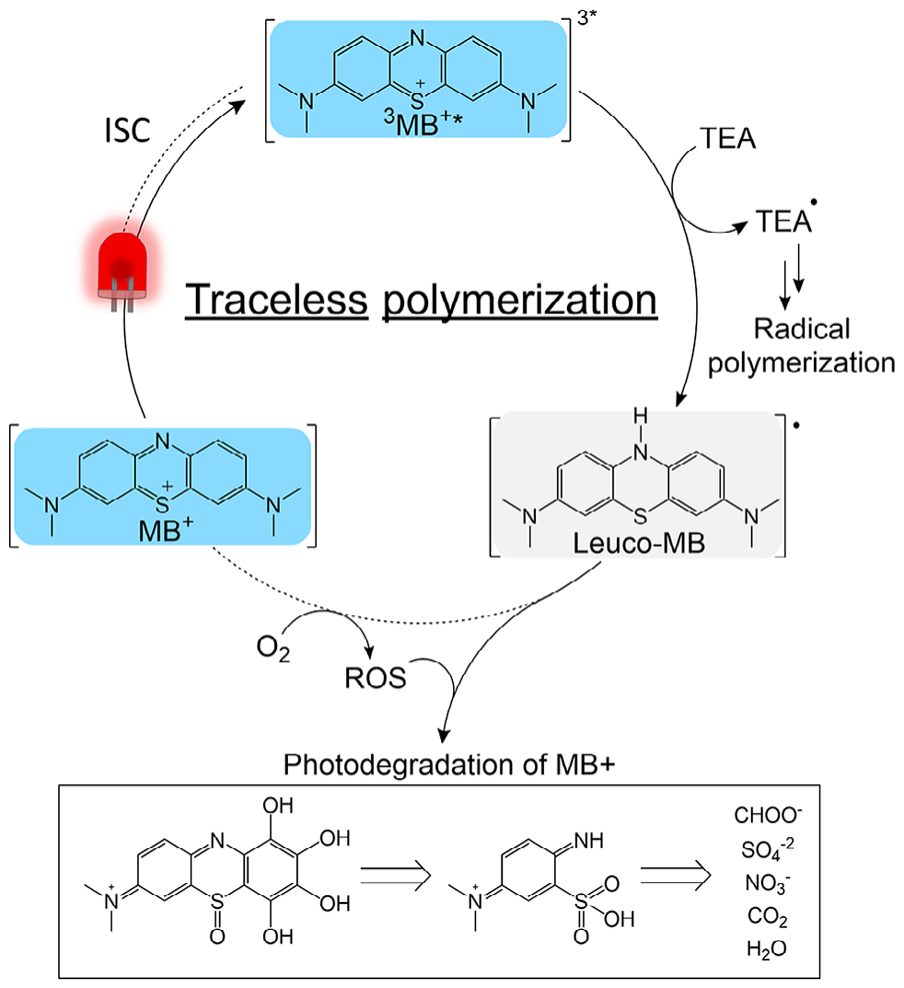
Photocatalytic cycle of MB^+^ with red light, TEA, and O_2_, producing both polymerization‐initiating species and self‐degrading species.

TEA was utilized as a sacrificial electron/proton donating agent (E_1/2_ (TEA/TEA^•^) = +0.61 V vs SCE),^[^ ^25^ ^]^ reacting with the ^3^MB^+*^ (E_1/2_ (^3^MB^+*^ /MB^•^) = +1.6 V vs SCE),^[^ ^21^ ^]^ to generate Leuco‐MB through the PET process (Equation (2)). The reduction of ^3^MB^+*^ to Leuco‐MB has been previously reported to proceed via 1e^−^/1H process, resulting in the formation of TEA^•^ radicals (**Figure** **2**).^[^ ^26^, ^27^ ^]^ TEA^•^ radicals are reactive toward DMA monomers and thus initiate polymerization. In a control experiment without TEA as a reducing agent, no photobleaching occurred.

Since the PET process occurs under ambient conditions, another potential reaction for ^3^MB^+*^ or Leuco‐MB involves quenching with ^3^O_2_, leading to the formation of several products (in an aqueous environment) as follows:

(2)

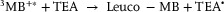



(3)

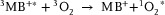



(4)

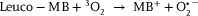

(5) $$O_{2}^{•-}+H^{+}\;\to {\mathrm{HO}}_{2}^{•}\;$$

(6) $$2{\mathrm{HO}}_{2}^{•}\;\to \;H_{2}O_{2}+O_{2}\;$$

(7) $$H_{2}O_{2}+e^{-}\left(\mathrm{Leuco}-\mathrm{MB}\right)\;\to \;{\mathrm{OH}}^{•}+\;{\mathrm{OH}}^{-}$$

Molecular oxygen (^3^O_2_) reacts with Leuco‐MB to regenerate MB^+^, which can then undergo another catalytic cycle for the generation of polymerization‐inducing radicals (Figure 2). Additionally, ^3^O_2_ competes with TEA to react with ^3^MB^+*^ (Equation (3)); ultimately, in both processes, ROS such as ^1^O_2_ and OH• (Equation (7)) are generated. These species eventually result in the permanent degradation of MB^+^. Chen et al. have investigated the degradation mechanism of MB^+^ in water upon visible light irradiation, focusing on wastewater treatment.^[^ ^28^ ^]^ This study showed that during irradiation and under ambient conditions, the initial degradation of MB^+^ primarily involves the cleavage of bonds in the dimethylamino group of MB^+^, likely attacked by singlet oxygen (^1^O_2_). This dissociation process eventually results in the complete disintegration of the benzene ring, as identified through high‐resolution liquid chromatography‐tandem mass spectrometry. Moreover, Sharma et al. investigated the photocatalytic degradation of MB^+^ via OH• under visible light irradiation aimed at wastewater purification.^[^ ^29^ ^]^ This study elucidates that OH• initiates the degradation of MB^+^ by substituting methyl groups with formyl groups and the cleavage of bonds within the MB^+^ molecule. Hence, using MB^+^ as a photoinitiator in plain water and under ambient conditions can offer the advantage of catalytically generating radical species while simultaneously sacrificing the photoinitiator itself. We leveraged the sacrificial nature of the photoinitiator for traceless photopolymerization, resulting in the production of a final colorless polymer under a range of power densities from 0.9 to 44 mW cm^−2^ upon red light irradiation.

### 2.1 Photopolymerization Kinetics

**Figure 3:**
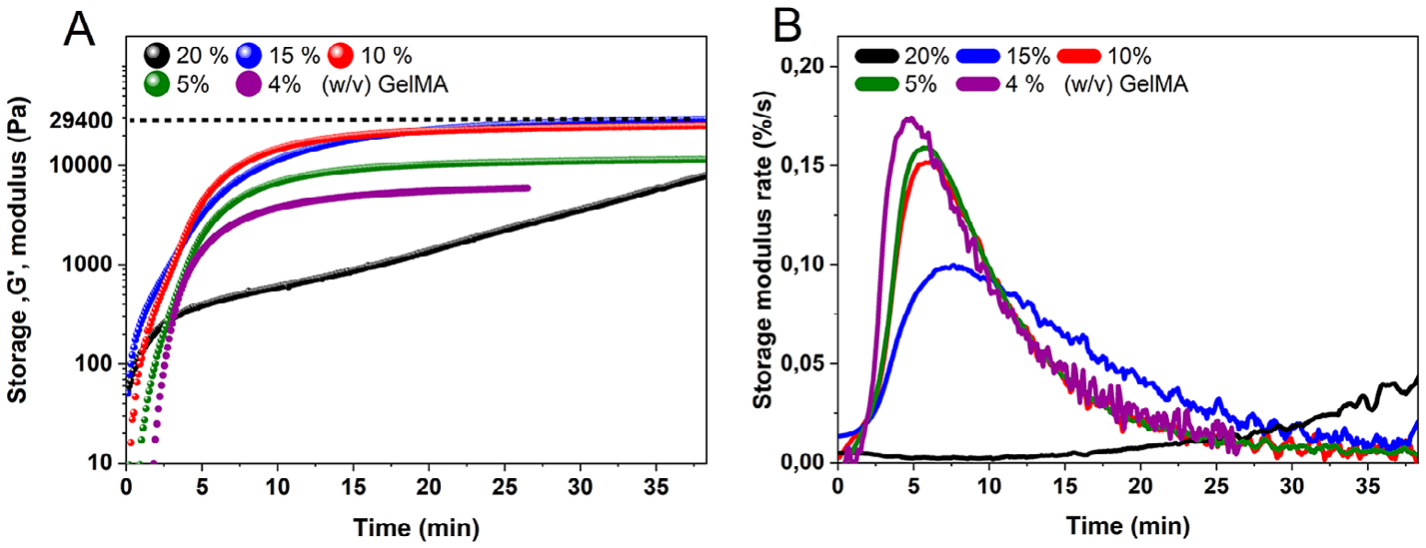
A) In situ photorheology measurements of GelMA formulations (4%, 5%, 10%, 15%, and 20%) to assess the gelation times. MB^+^, TEA, and DMA concentrations were constant for all formulations at 16 µm, 37 mm, 4 v/v %, respectively (time sweep of oscillatory measurement, 44 mW cm^−2^ for 30 min, at 20 °C). B) The derivative of the storage modulus with respect to time is used to show the relative storage modulus rate.

To reveal the reaction kinetics and mechanical characteristics during ambient photopolymerization, in situ photorheology was employed. First, different concentrations of GelMA (4%, 5%, 10%, 15%, and 20% w/v) were analyzed to determine the influence of initial viscosity on polymerization kinetics under ambient conditions (*T* = 20 °C).^[^ ^30^ ^]^ In free radical polymerization, the propagation rate is often diffusion‐controlled; thus, an increase in GelMA concentration, which leads to higher viscosity, can hinder the diffusion rate and potentially affect the polymerization rate.^[^ ^31^, ^32^, ^33^ ^]^ Upon initiating irradiation at 625 nm (44 mW cm^−2^), photopolymerization started immediately in GelMA samples with concentrations of 20%, 15%, and 10% (**Figure** **3**A), resulting in a gelation time of 0 s. Gelation time defined as the point at which the storage modulus (G′) exceeded the loss modulus (G″), as shown in Figure S2A–C (Supporting Information). In contrast, for the 5% and 4% GelMA concentrations, there was a delay of ≈50 and 96 s, respectively, before photocrosslinking started (Figure S2D,E, Supporting Information). This delay could be attributed to the lower polymer concentration requiring a longer time to reach the critical crosslinking density necessary for gelation. Additionally, increased oxygen diffusion in the sample leads to higher quenching of ^3^MB^+^*, which leads to the production of ^1^O_2_^27^, as shown in Equation (3). In addition, the produced ^1^O_2_ can react with triethanolamine, which leads to its degradation.^[^ ^34^, ^35^ ^]^ Both of these processes hinder polymerization initiation, resulting in a longer lag phase.

Furthermore, up to a GelMA concentration of 15%, there is a direct correlation between increasing GelMA concentration and higher plateau values of the storage modulus (G′) (Figure 3A). The samples with 15% GelMA achieved the highest G′ (≈29 kPa) within 30 min. However, at 20% GelMA, the samples did not reach a plateau within the same time frame, resulting in a lower G′ than with the other concentrations. One possible explanation is that at very high GelMA concentrations, local inhomogeneities or partial phase separation may occur, preventing the formation of a uniformly crosslinked network. Additionally, the rate‐of‐change of storage modulus over time, expressed as a percentage per second (%/s), is derived from the first‐order differentiation of the normalized G′ values shown in Figure 3B.^[^ ^36^ ^]^ This determines how quickly the hydrogel network stiffens at any given moment. The peak in the storage modulus rate of change for each GelMA concentration corresponds to the most active phase of polymer network formation. The subsequent decline indicates a slowing of the cross‐linking process as the network approaches its final stiffness. It is observed that the storage modulus rate of change decreases as the GelMA concentration increases. Notably, 4% GelMA displays the steepest peak in the rate of change curve once polymerization initiates. In contrast, higher GelMA concentrations (e.g., 10% or 15%) generally start crosslinking sooner but exhibit a lower maximum rate of change.

The storage modulus for GelMA concentrations of 4%, 5%, 10%, and 15% reached a plateau within ≈10 min, which is comparable to the times observed in other studies that utilized MB^+^ as a photoinitiator (Figure 3A).^[^ ^23^, ^24^ ^]^ Importantly, for GelMA, concentrations ranging from 4% to 15%, G′ exceeds 10^3^ Pa within the first 5 min, suggesting rapid gelation of the sample. Moreover, increasing the GelMA concentration to 15% reduced the calculated average mesh size (Equation (8)), thereby increasing the cross‐linking density (Equation (9)) and stiffness of the hydrogels (Figure S3, Supporting Information). However, despite this higher GelMA concentration, the average mesh size and cross‐linking density of the 15% GelMA hydrogels did not show significant improvement compared to the 10% GelMA hydrogels.

**Figure 4:**
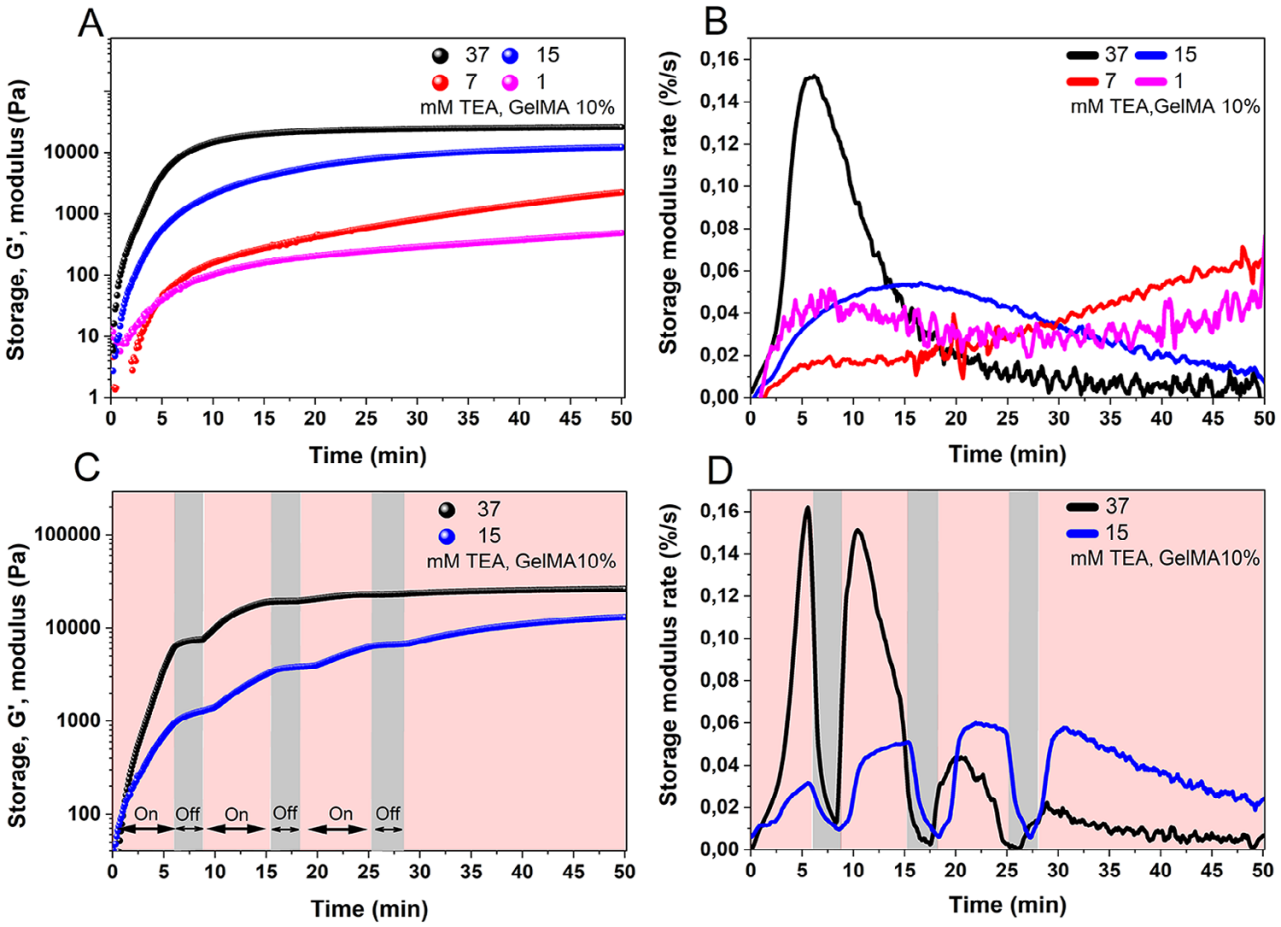
A,B) The effects of different concentrations of TEA on the storage modulus and the storage modulus rate of change, respectively. C) Temporal control of photopolymerization through multiple on/off cycles of red light during in situ photorheology measurements. D) The impact of on/off cycles of the red light on the storage modulus rate of change. All measurements were conducted in GelMA 10% under 625 nm light irradiation (44 mW cm^−^^2^). MB^+^ and DMA concentrations were constant for all formulations at 16 µm and 4 v/v %, respectively.

To improve the biocompatibility of the photopolymerization process, one important factor is to minimize the concentration of the co‐initiator. Several previous studies have examined the cytotoxicity of TEA.^[^ ^37^, ^38^ ^]^ These studies employed TEA as a co‐initiator across a range of concentrations, for example, up to 450 mm, and reported cell viability values of more than 80%. In the present study, we examined the effect of TEA concentration, ranging from 1 to 37 mm, on the relative reaction rate at 44 mW cm^−2^. As TEA concentration increases, a corresponding decrease in both gelation and lag time is observed, along with an increase in the storage modulus (**Figure** **4**A). This observation is partially explained by Equations (2) and (3), which predict a competitive reaction between oxygen and TEA with MB^+^ as reported by Chaberek et al.^[^ ^39^ ^]^ At higher TEA concentrations, the formation of TEA^•^ radical becomes predominant, resulting in an enhanced polymerization rate, as evidenced by a higher storage modulus rate of change (Figure 4B). Moreover, the rate of change increases notably when the TEA concentration exceeds 15 mm. This indicates that at higher concentrations (e.g., 37 mm), TEA successfully competes with ground‐state oxygen to interact with ^3^MB^+^* for efficient photoinduced electron transfer (Equations (2) and (3)). Simultaneously, TEA scavenges the singlet oxygen generated during the photopolymerization process. As a result, this TEA concentration is enough to sustain fast photopolymerization kinetics even under ambient conditions.

In light‐assisted radical polymerization, radicals are continuously produced during irradiation, and as soon as the energy supply (light) is removed, the radical‐initiated reactions quickly stop. Therefore, by turning the red light on and off, a spatiotemporal control over the polymerization can be achieved, as demonstrated by the development of the storage modulus upon intermittent irradiation. Thus, the stiffness of the hydrogels, which is an important property for various applications such as cell culturing, can be tailored through the adjustment of irradiation time.^[^ ^40^, ^41^, ^42^ ^]^ To demonstrate temporal control within this system, we performed on/off cycles of red‐light irradiation (Figure 4C). When the light was turned off, the storage modulus remained constant, indicating that no further polymerization occurred. Accordingly, the storage modulus rate of change quickly decreased when the light was turned off and increased rapidly as soon as the light was turned back on (Figure 4D). By comparing the final storage modulus at two different concentrations of TEA (15 and 37 mm in Figure 4A,C, respectively) and comparing the storage modulus and storage modulus rate of change (Figure 4B,D), it can be concluded that multiple on/off cycles of red light did not adversely affect the final mechanical properties and the kinetics of polymerization. To demonstrate spatial control, a test tube filled with the ink was partially irradiated (Figure S4A–C, Supporting Information), resulting in a colorless region only where the light was applied. Additionally, when a photomask was applied and irradiated, only the exposed area crosslinked and became colorless (here, the Tampere University logo was used), confirming successful spatial control of the system (Figure S4D–G, Supporting Information).

To further demonstrate the versatility of our red‐light‐initiated photopolymerization system (MB⁺/TEA), we tested its applicability to polyethylene glycol diacrylate (PEGDA, *M*_n_ = 750 g mol^−1^), another widely used hydrogel system. As shown in Figure S5 (Supporting Information), 50% v/v PEGDA solution underwent immediate gelation upon irradiation (625 nm, 44 mW cm^−^^2^), reaching a final storage modulus above 10 kPa. These results highlight the broad applicability and robust performance of our photoinitiation system across different hydrogel platforms.

To utilize this photocuring system in biological applications, the effect of cell culture media, such as DMEM, on polymerization kinetics should be investigated. In addition, lower GelMA concentrations (<5% w/v) are preferred as they offer a less viscous medium that mimics the natural extracellular matrix, enhancing nutrient and oxygen diffusion and promoting cell viability and proliferation. However, these low concentrations impact the mechanical properties and printability in processes such as extrusion 3D printing. To achieve good printability, the temperature of GelMA‐based ink must be cooled down during printing (e.g., to 17 °C). Therefore, to compare the reaction kinetics and mechanical properties of 4% GelMA in different media and at various temperatures, photorheological measurements were performed in DMEM over a temperature range of 4 to 37 °C (Figure S6, Supporting Information). In general, cell culture media ingredients can interfere with radical polymerization by radical scavenging and/or radical chain transfer effects.^[^ ^43^ ^]^ However, when using DMEM as a reaction medium at 17 and 20 °C, no significant effect on the kinetics and final mechanical properties was observed when compared to results obtained from water at 20 °C (Figure S6, Supporting Information). At 37 °C, the polymerization proceeded faster and reached its plateau earlier, yet the final storage modulus remained nearly the same as for other temperatures. Interestingly, at 4 °C, although the polymerization exhibited slower kinetics, both the initiation point, and the final storage modulus were comparable to those observed at higher temperatures.

**Figure 5:**
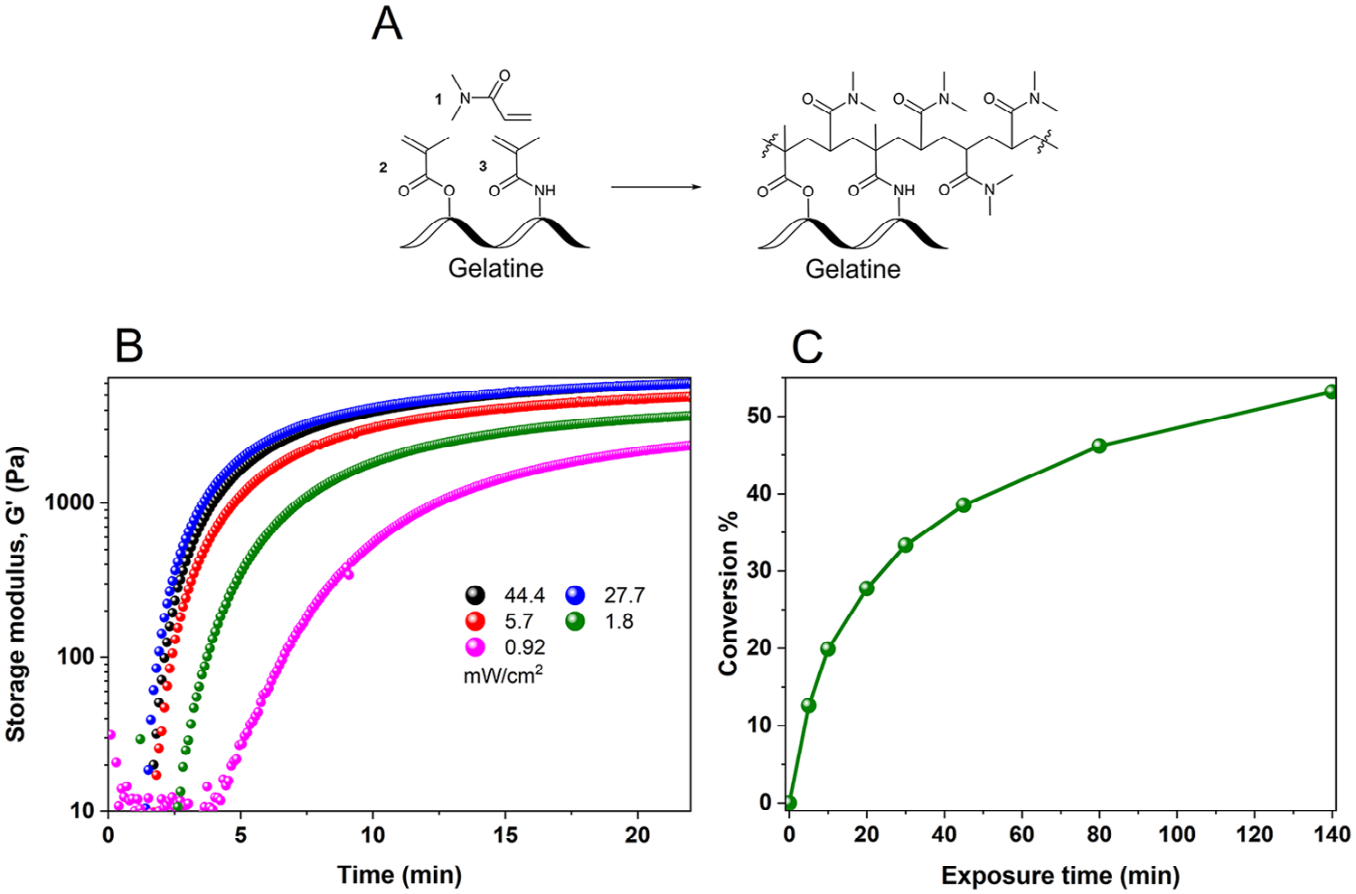
A) Photopolymerization of dimethylacrylamide and methacryloyl‐derivatized gelatine. B) Power density effect on the kinetics of polymerization. Increasing the power density from 0.9 mW cm^−2^ to 5.7 mW cm^−2^ results in a significant effect on the final storage modulus, kinetics, and delayed time. After 5.7 mW cm^−2^, the improvements are not significant. The measurements were performed in 4% w/v GelMA, 37 mm TEA, and 4% v/v DMA in DMEM buffer. C) In situ ^1^H‐NMR spectroscopy measurements of monomer conversion in a photopolymerization reaction. The measurements were conducted on a solution containing 4% w/v GelMA, 16 µm MB, 37 mm TEA, and 4% v/v DMA in deuterated water, with a power density of 30 mW cm^−^^2^ at a wavelength of 625 nm.

The effect of power density (0.9–44 mW cm^−^^2^) on the kinetics of photopolymerization was investigated in a 4% GelMA solution (**Figure** **5**B). In general, increasing power density results in faster kinetics, improvement of the final storage modulus, and shorter lag time. Initially, increasing the power density to 5.7 mW cm^−^^2^ significantly enhances the rate of polymerization, final storage modulus, and reduces lag time. However, beyond this point, further increases in light intensity do not yield significant improvements in either kinetics or final storage modulus.^[^ ^32^, ^44^ ^]^ This phenomenon, the effect of light intensity on the kinetics of free radical polymerization, was explained by Luu et al.: up to a certain light intensity level, the radical polymerization rate depends on the square root of the light intensity.^[^ ^45^ ^]^ It has been reasoned that higher light intensities generated large concentrations of short macroradical chains, which primarily facilitate bimolecular termination and potentially limit further enhancements in polymer properties.^[^ ^46^ ^]^

To directly assess the proposed red‐light‐based method's enhancement of the mechanical strength of GelMA hydrogels, compared to traditional UV or blue‐light curing methods, we performed comparative photorheology experiments. Under identical conditions (GelMA 4%, 40 mW cm^−^^2^ irradiation), the storage modulus (G′) of hydrogels crosslinked using our red‐light approach (MB⁺/TEA system) reached ≈2.8 kPa. In contrast, hydrogels cured with blue light (using 0.1% LAP photoinitiator) exhibited faster initial crosslinking kinetics but reached a substantially lower plateau storage modulus (≈0.5 kPa, Figure S7, Supporting Information). These results clearly demonstrate that the proposed red‐light photocuring method significantly improves the final mechanical strength of GelMA hydrogels compared to traditional UV/blue irradiation methods.

Monomer conversion in a GelMA 4% w/v solution was quantified using ^1^H‐NMR spectroscopy. It gives an insight into the formation of the network and evaluates the efficiency of polymerization (Figure 5). It should be noted that the composition used for polymerization contained three different acrylic groups (Figure 5A): free dimethylacrylamide 1; methacrylic esters 2 at hydroxyprolines and hydroxylysines of gelatine; methacrylic amides 3 at lysines and arginines of gelatine. All three types of acrylic double bonds produce the ^1^H NMR peaks in a narrow region 5.5–6.5 ppm, and their signals overlap. However, in the molar ratio used here, the signals of DMA at 6.55, 5.95, and 5.6 ppm dominate by a large margin, and therefore were chosen for quantitative evaluation of polymerization. Detailed assignments of these signals, their changes during irradiation, and respective conversion calculations are provided in the Supporting Information (“NMR Spectroscopy section”, Figures S19–S22, Supporting Information). The progression of the photopolymerization reaction was followed upon exposure to the red light (625 nm, 30 mW cm^−2^). In the first 10 minutes, a rapid decrease in intensity of the double bonds’ signals and a high monomer conversion (near 20%) are observed. After 130 min of irradiation, the quantity of methacrylate double bonds reaches a plateau, achieving over 50% monomer conversion. Subsequently, the conversion percentage increases very slowly, if at all. This phenomenon is attributed to the high crosslinking density, which hinders the diffusion of macroradicals and thereby slows the monomer conversion process. Additionally, the reaction may also decelerate due to the degradation of MB^+^, a process that occurs through its interaction with singlet oxygen and OH•, further reducing the polymerization rate. Monomer conversion in a 10% w/v GelMA solution was also quantified (Figure S8, Supporting Information). The data show that the monomer conversion is fastest during the first 30 min of continued irradiation, after which it slows down and reaches a plateau at ≈48% conversion at 80 min. This result aligns with the storage modulus measurements for a solution of GelMA 10% with a 15 mm TEA concentration (Figure 4A, blue color). Within the first 30 min, the storage modulus rises to 10 kPa, indicating a high cross‐linking density, which restricts radical diffusion and slows monomer conversion.

### 2.2 Red‐Light Induced Photopolymerization through Biotissue

In tissue engineering, injectable, photoresponsive bioinks can hold great promise for non‐invasive tissue reinforcement and reconstruction.^[^ ^47^ ^]^ To evaluate the performance of our photocuring system, three pieces of 5% agar gel were prepared as tissue mimics, designed to simulate light‐tissue interactions in human tissues.^[^ ^48^, ^49^ ^]^ Interestingly, photorheology measurements showed no difference in the kinetics of the photocuring system or final storage modulus of the GelMA gel between thicknesses of 1 and 5 mm, despite their differing transmittance (Figure S9, Supporting Information). These results align with the power density measurements, where increasing the light intensity did not significantly affect the kinetics or the final storage modulus after reaching a plateau value.

**Figure 6:**
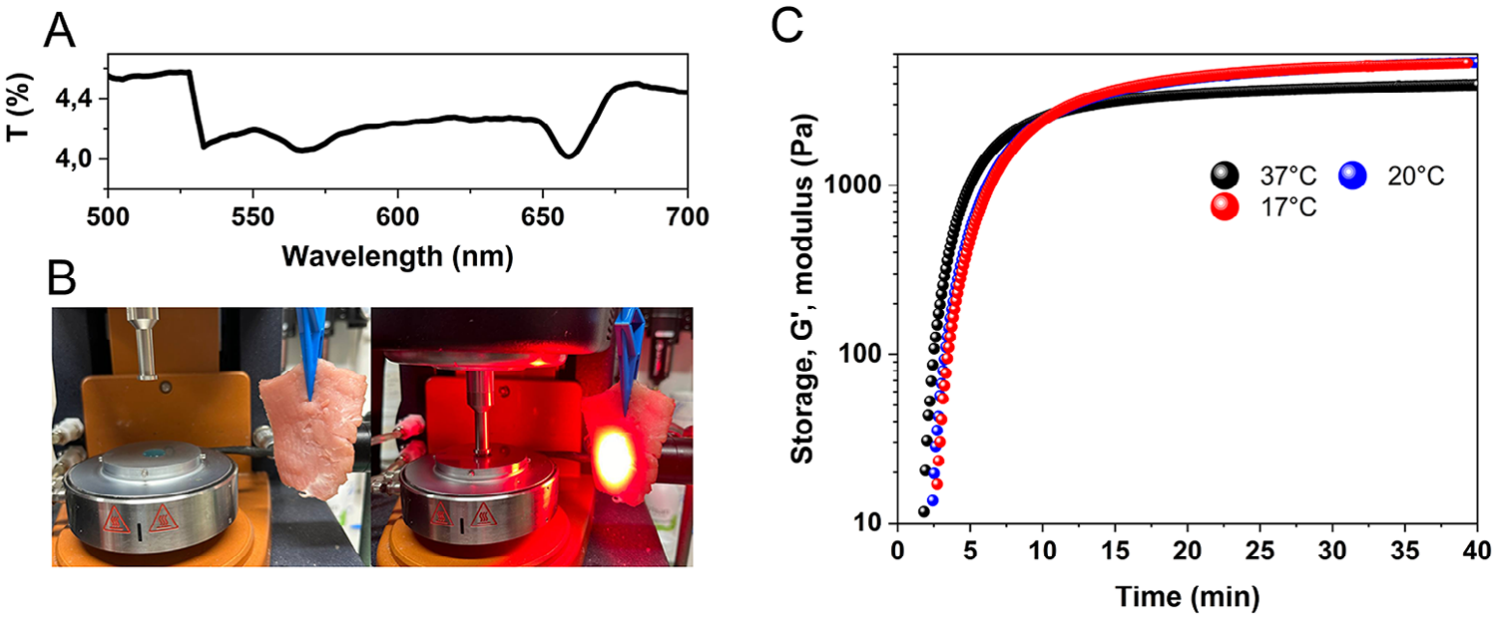
Photopolymerization through chicken breast with a thickness of 4.8–5 mm upon red light irradiation. A) Light transmittance measurement of a chicken breast piece shows only 4.2% of light can penetrate through it. B) A chicken breast piece was positioned in front of the LED for photorheology measurement. C) In situ photorheology measurement in the presence of a piece of chicken breast. All measurements were conducted in GelMA 4% w/v, 16 µm MB, 37 mm TEA, and 4% v/v DMA under initial irradiation of 44 mW cm^−^^2^ at 625 nm.

In addition, the performance of the photocuring system was also tested with a chicken breast as a tissue phantom. At first, the light transmittance spectrum of a chicken piece (thickness: 4.9 ± 0.1 mm) was measured, showing that ≈4.2% of the light could pass through it (**Figure** **6**A). As shown in Figure 6B,C, the measurements were conducted at three different temperatures to compare the performance of the photocuring composition when used in cooled‐head extrusion‐based 3D printing and as an injectable gel at physiological body temperatures. Increasing the temperature to 37 °C initially boosts the kinetics by accelerating the radical propagation rate yet leads to a lower terminal storage modulus, although these changes are not significant. It is worth mentioning that across all temperatures, the storage modulus reaches 1 kPa in less than 8 min, demonstrating its potential for use in‐depth curing applications. In addition, the same measurement was performed with GelMA 10% for the three different pieces at the same temperature (Figure S10, Supporting Information). For three pieces of chicken (thickness: 4.9 ± 0.1 mm), the average storage modulus of the gels reached a plateau of 9.6 kPa within 30 min, and the color disappeared from the gels. This measurement shows that a high storage modulus can be achieved for in‐depth curing applications by using high GelMA concentration.

### 2.3 3D Printing Feasibility Testing

In general, the printability of bioink in extrusion‐based printing can be assessed by various methods, which consider the extrudability, filament fidelity, and scaffold design‐related factors.^[^ ^50^ ^]^ It is feasible to start the evaluation by checking the flow behavior of the bioink, followed by assessing the uniformity of printed filaments and pores, and finally, measuring the overall accuracy of the printed scaffold in comparison to the original design.^[^ ^51^ ^]^ Both inks behaved quite similarly and were able to form continuous filaments with relatively smooth surfaces (Figure S11, Supporting Information). Furthermore, GelMA 10% w/v formed somewhat longer filaments than GelMA 4% w/v.

**Figure 7:**
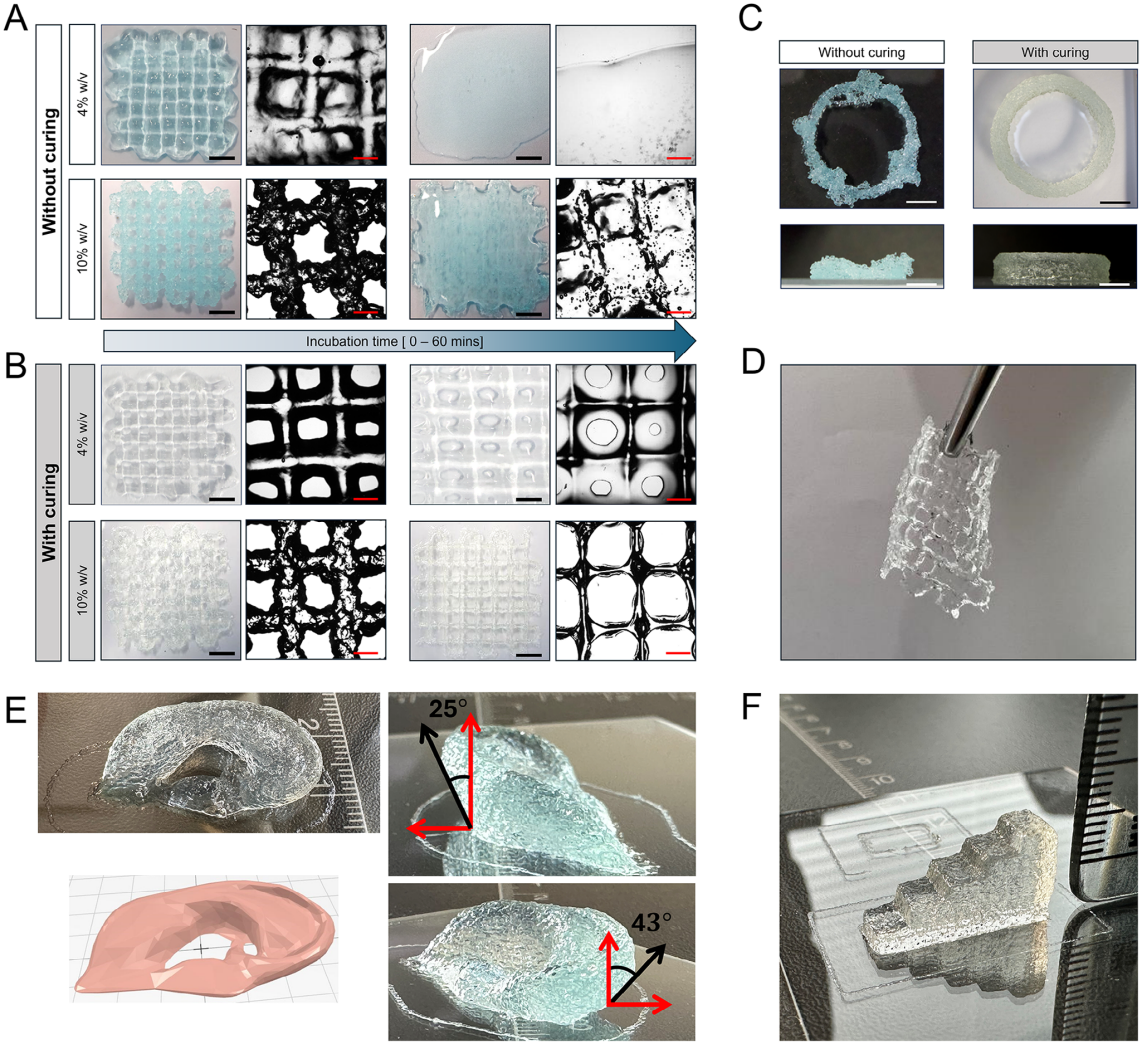
The printability and stability of GelMA at 37 °C were examined under various conditions: polymer concentrations (4% and 10% w/v) and photocuring (with and without). A) 4 and 10% w/v of GelMA without photocuring B) 4% and 10% w/v of GelMA without photocuring. C) Top and side views of printed cylinders using 10% w/v of GelMA with and without photocuring. D) Printed GelMA grid structures held by a tweezer. E) Miniaturized ear model printed from GelMA 10% w/v at RT. F) Staircase model printed from GelMA 10% w/v at RT. Ink formulation: 16 µm MB, 37 mm TEA, and 4% v/v DMA. Scale bars: 5 mm (overall) and 0.125 mm (microscope).

To pre‐evaluate the printability of our traceless polymerizable bioinks, GelMA 4% w/v and 10% w/v were printed into 6‐layered grid structures via extrusion‐based printing. To assess the influence of printing conditions on the printability and stability of printed constructs, the samples were divided into two categories: printed with and without curing (**Figure** **7**A,B).

According to the literature, GelMA hydrogels with low concentrations (≤5% w/v) provide better cell cytocompatibility and viability. However, the processability and structural stability are poor because of low viscosity, slow gelation time, and low mechanical strength.^[^ ^52^, ^53^ ^]^ In this study, 4% w/v GelMA was printed at 17 °C to obtain good printability and stability during and after printing without interfering with the gelation time of hydrogel during photocuring. This strategy has been widely used due to the thermoresponsive properties of gelatine.^[^ ^51^, ^54^ ^]^ As expected, 4% w/v GelMA without in situ curing exhibited poor printability and displayed low stability after printing compared to the photocured samples, exemplified by deposited filaments merging together (Figure 7A). Although increasing polymer concentration improved the printability (without curing), the stability of uncured structures remained poor, leading to liquefying after 60 mins of incubation at 37 °C. In contrast, the printed structures with photocuring had better stability under incubation due to the formation of covalently crosslinked networks (Figure 7B). After incubation, the microscopic images revealed that the cured structures had shrunk and were uniformly thinner. However, the filaments remained intact without detachment or spreading, regardless of whether they were printed using 4% or 10% w/v GelMA.

These results demonstrate that our in situ photocuring setup provides an effective approach for the 3D printing of low‐concentration polymers while maintaining precise dimensional accuracy and shape retention during and after printing. During printing, the non‐viscous ink undergoes simultaneous photocuring to form a uniform printed filament. This process also enhances the cohesion between layers, resulting in greater stability. The stability is further evidenced by the structure's resistance to breakage when handled with tweezers (Figure 7D). Moreover, the color of the photocured constructs changed from light blue to colorless, confirming again the successful photodegradation of methylene blue within the polymer network.

After optimizing the printing conditions, GelMA 4% w/v and 10% w/v were printed into multilayered cylinders (OD = 20 mm, *h* = 4.2 mm) to evaluate the shape fidelity of 3D constructs. Furthermore, GelMA 10% w/v was also printed into miniaturized ear (Figure 7E, *W* = 15.99 mm, *L* = 32 mm, *H* = 6.87 mm) and staircase (Figure 7F; Figure S14, Supporting Information, *W* = 6.25 mm, *L* = 18.84 mm, *H* = 9.38 mm) models to showcase the ink's ability to maintain shape fidelity in more complicated geometries with varying z‐slices. The effect of printing without in situ curing versus with curing was further showcased by printing cylinders from 10% w/v of GelMA (Figure 7C). The cohesion between layers of deposited filaments during in situ photocuring resulted in the formation of complete circular printed shapes with straight walls, indicating high structural integrity.

The dimensions (wall height, outer and inner diameters) of printed cylinders were measured and compared to the theoretical dimensions of the CAD model to evaluate the shape fidelity (Figure S12, Supporting Information). As shown in Table S1 (Supporting Information), both inks produced cylinders with high shape fidelity and optimal layer stacking, that is, having shape fidelity indices close to 1 (e.g., 0.97 for 4% w/v GelMA cylinder wall height and 1.14 for 10% w/v GelMA cylinder wall height). However, the inner diameters of both cylinders deviated from the theoretical diameter considerably and resulted in shape fidelity indices of <1. This is likely due to the quite prominent filament swelling after being extruded from the nozzle, which contributed to the wall thickness of the cylinders and diminished the inner diameter. Two of the overhang angles of the printed ear (Figure 7E) were also measured and compared with the corresponding CAD model (Figure S13, Supporting Information). The backside of the printed ear featured an overhang of ≈65° (compared to ≈62° in the CAD model), while the top of the ear had an overhang of ≈47° (compared to ≈49° in the CAD model). Hence, the dimensional analysis of the staircase model and the ear model printed from GelMA 10% w/v further supported the observation of the ink's excellent shape fidelity (Table S2, Figure S14, Supporting Information). Overall, both GelMA ink concentrations showed excellent printability and shape fidelity. However, the 4% w/v GelMA appears to produce a smoother surface topology than 10% w/v GelMA, which probably results from 10% w/v GelMA being a more viscous ink. Hence, a GelMA ink with a lower concentration would be preferred for bioprinting applications with living cells.

### 2.4 Cytocompatibility of the Formulation

Another important requirement for a hydrogel to be utilized as a bioink is cytocompatibility. Among the different components of the GelMA formulation, unsurprisingly, DMA was found to be the most toxic. NIH­3T3 fibroblasts incubated with 4% v/v DMA dissolved in a complete medium for one hour at 37 °C could not recover and died (data not shown). Thus, 4% w/v GelMA hydrogels with 1 or 4% v/v DMA were compared (Figure S15, Supporting Information). After curing with red light for 10 minutes at room temperature, the bioink containing 1% v/v DMA showed higher cell viability than with 4% v/v DMA, with 50% vs. 32% viability, respectively (Figure S15A,B, Supporting Information). Moreover, when cells were incubated for 30 minutes with the 4% v/v DMA formulation prior to curing, the percentage of living cells dropped to 18% (Figure S15A, Supporting Information). However, no incubation‐dependent toxicity was observed for the 1% v/v DMA formulation (Figure S15B, Supporting Information).

Keeping cells at low temperatures (0–4 °C) is a common approach to preserve their viability for a few hours.^[^ ^55^ ^]^ Notably, the NIH‐3T3 cells successfully recovered and proliferated after being incubated with 4% v/v DMA for one hour at 4 °C (data not shown). Therefore, the impact of curing the bioink at 4 °C on the cell viability was investigated. GelMA (4% w/v) bioinks with 4% v/v DMA were treated with red light either at room temperature or 4 °C. On day 2 in culture, the cell‐laden hydrogel crosslinked at 4 °C showed about two times higher viability than the one crosslinked at room temperature (Figure S15C, Supporting Information).

**Figure 8:**
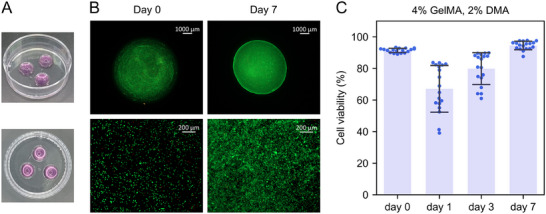
The viability of NIH‐3T3 fibroblasts in 4% w/v GelMA hydrogel (16 µm MB, 37 mm TEA, and 2% v/v DMA). A) The bioink was shaped in the form of a drop and treated with red light for 10 min at 4 °C. B) Representative images showing live (green) and dead (red) cells in the crosslinked hydrogel immediately after the red‐light treatment (day 0) and after culturing for 7 days (day 7). The cells were stained with FDA/PI. Scale bars: 1000 µm (upper row) and 200 µm (bottom row). C) The percentage of live cells over time. Two independent experiments were done with *n* = 3 per every time point. Three images were taken per sample, resulting in 18 images per every time point. The data is shown as mean ± SD; each dot represents an image.

These results indicate that lowering the DMA concentration and curing the bioink at 4 °C improves the cytocompatibility of the formulation. Also, since the monomer conversion in 4% w/v GelMA solution after 10 min of red‐light treatment is ≈20%, an additional washing step might be beneficial to reduce the amount of potentially toxic unreacted species. Taking together the described considerations, the viability of NIH‐3T3 fibroblasts embedded into the 4% w/v GelMA with 2% v/v DMA hydrogel was monitored over the course of seven days. (**Figure** **8**). The cell viability was >90% right after the red‐light treatment. However, some delayed toxicity was observed on day 1 when ≈65% of cells remained viable (Figure 8C). After day 1, the cells started to recover and adhered to the crosslinked GelMA material, reaching >95% viability by day 7.

A formulation of 4% w/v GelMA can be easily printed at 17 °C. However, as a lower temperature increases the viscosity of GelMA inks, it becomes difficult to print at 4 °C, which is the desired handling temperature for the cells. Hence, a new printing protocol was established: To ensure a printable viscosity at this temperature, the GelMA concentration in the ink was lowered. With 3% w/v GelMA hydrogel, prints achieved a comparable resolution and stability (Figures S16 and S17, Supporting Information). An investigation of the curing kinetics of 3% w/v GelMA ink at 4 °C showed that lowering the DMA concentration to 2% had a minor effect on the kinetics, while lowering the GelMA concentration from 4 to 3% did slow the kinetics significantly. However, within 15 min, a storage modulus of ≈1 kPa was reached (Figure S18, Supporting Information).

### 2.5 3D Bioprinting Followed by Photopolymerization

**Figure 9:**
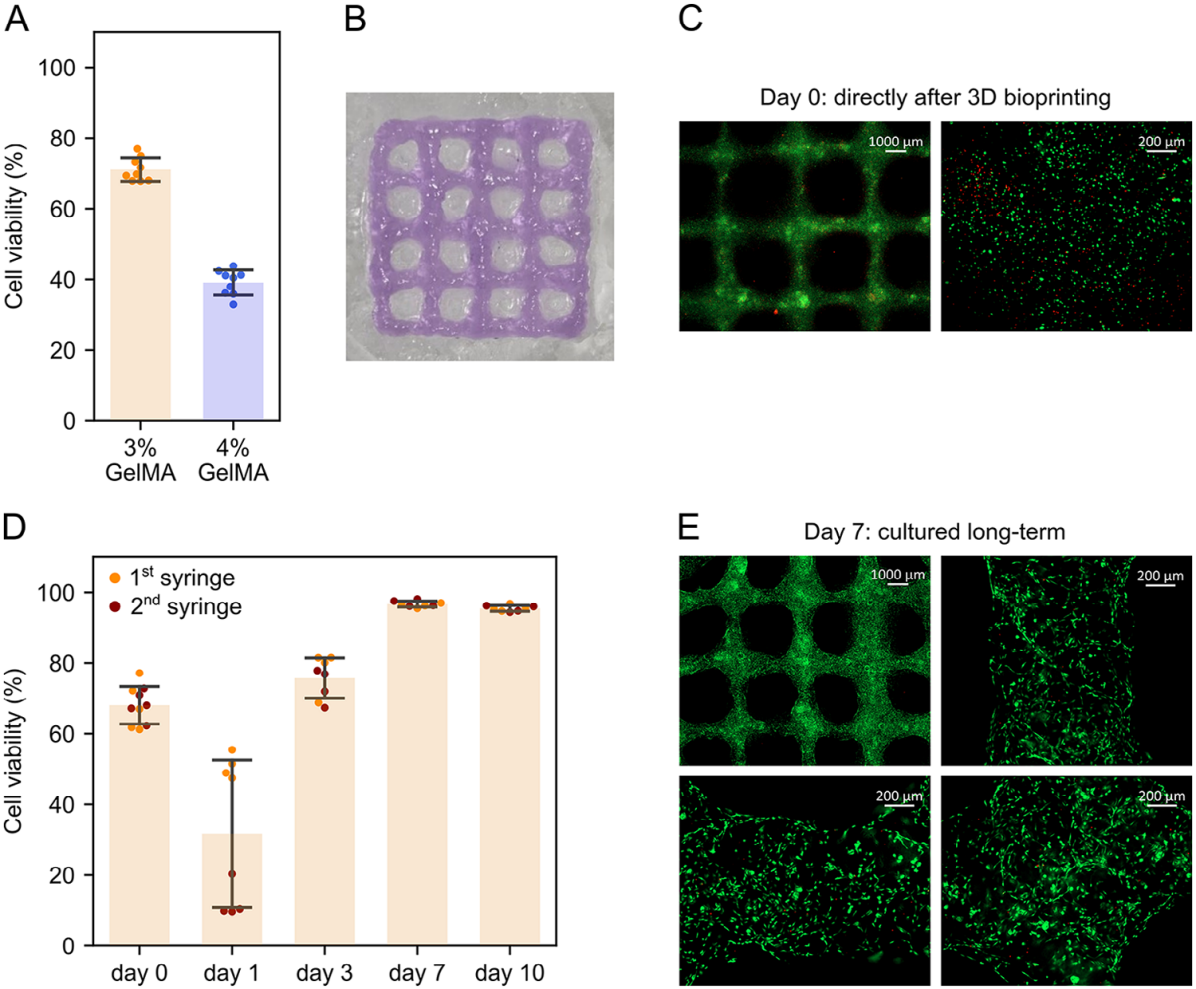
NIH‐3T3 fibroblasts 3D bioprinted with GelMA hydrogels. The cells were mixed with 4 or 3% w/v GelMA (16 µm MB, 37 mm TEA, and 2% v/v DMA) hydrogel and 3D bioprinted at 17 or 4 °C, respectively. The grids were photocured for 10 min and washed twice (all at 4 °C). A) The percentage of living cells in 3 or 4% GelMA grids (*n* = 3) directly after the 3D bioprinting and red‐light treatment. The data is shown as mean ± SD; each dot represents an image. Three images were taken per sample, resulting in 9 images per formulation. B) Top view of the 3D bioprinted grid using 3% GelMA. C,E) Representative images showing live (green) and dead (red) cells in the photocured 3% GelMA hydrogel directly after the 3D bioprinting (day 0) and after culturing for 7 days (day 7). The cells were stained with FDA/PI. Scale bars: 1000 and 200 µm. D) The percentage of living cells over time. One grid printed with the first syringe and one grid printed with the second syringe (*n* = 2) were used per time point. Four images were taken per grid, resulting in 8 images per time point. The data is shown as mean ± SD; each dot represents an image.

Two cytocompatible protocols were established for extrusion‐based 3D bioprinting using 4% w/v GelMA hydrogel at 17 °C or 3% w/v GelMA hydrogel at 4 °C. To determine which protocol is better suited for 3D bioprinting, NIH‐3T3 fibroblasts were mixed with the formulations. Each bioink was used to print grid structures (*n* = 3) at the respective temperatures, followed by red light curing at 4 °C. The cells printed with 3% w/v GelMA showed ≈70% viability, which was almost two times higher than the viability of the cells printed with 4% w/v GelMA (**Figure** **9**A). This difference in cell viability clearly revealed the importance of low temperature for 3D bioprinting with traceless polymerizable bioinks.

Proceeding with the 3% w/v GelMA formulation, the viability of 3D bioprinted grids was assessed over the course of ten days. In total, two syringes with 3% GelMA bioink were prepared. For both syringes, the NIH‐3T3 fibroblasts were harvested at the same time, resulting in a 3‐hour waiting time before preparing the second syringe. Ten grids were printed and used to measure the NIH‐3T3 fibroblast viability on days 0, 1, 3, 7, and 10 (Figure 9B–E, Supporting Information). Right after the red‐light treatment, the cells had a round shape, and their viability was ≈70% (Figure 9C). Notably, there was a clear difference in the cell viability between the grids from syringes 1 and 2 on day 1. The cells printed with the 1^st^ syringe maintained higher viability compared to the second syringe (Figure 9D). This demonstrates time‐criticality in harvesting and utilization of cells for 3D bioprinting.

Overall, the viability pattern observed after the 3D bioprinting resembled the previous long‐term study with a drop model (Figures 9D and 8C). The cells fully recovered by day 7 in culture and showed elongated morphology, suggesting they adhered to the hydrogel material (Figure 9E). Therefore, the 3% w/v GelMA (16 µm MB, 37 mm TEA, and 2% v/v DMA) formulation was found cytocompatible in extrusion‐based 3D bioprinting.

Summarizing our bioprinting experiments results, 10% w/v GelMA was printable at room temperature but required high extrusion pressure, which compromised cytocompatibility. GelMA at 4% concentration was not printable at room temperature but became printable at 17 °C. However, at this temperature, 4% DMA showed cytotoxicity during incubation. To improve cell viability, we explored incubation and printing at 4 °C. While 4% GelMA remained printable at this temperature (data not shown), it still required high extrusion pressures, which negatively impacted cell viability due to increased shear stress. To overcome this issue, we reduced the GelMA concentration to 3%, allowing extrusion at lower, cytocompatible pressures while maintaining printability and short gelling time.

### 2.6 Limitations

As exemplified before, the system is clearly applicable for different water‐soluble ink formulations and thus suitable for a wide range of (bio‐)printing applications. However, a few limitations can also be identified.

For the current bioprinting protocol, a low concentration of GelMA is required, which limits the mechanical strength of prints and consequently, the variety of models that can be printed.

Moreover, the need to perform bioprinting with chilled syringes, on chilled beds, and cure in a temperature‐controlled environment adds some complications to the (bio)printing process. These low‐temperature conditions are required to solve the issues of 1) fast cellular uptake of unreacted DMA and 2) printability of GelMA bioinks (vide supra). The first issue can be tackled by using a more cytocompatible monomer to substitute DMA. Meanwhile, the addition of sacrificial thermogelling (LCST) bioink will allow printing and curing GelMA compositions even at room temperature.^[^ ^56^ ^]^

It is noteworthy, since the polymerization process is initiated by photoinduced electron transfer, any change that would significantly hinder the PET (such as low polarity organic solvent) will inevitably diminish the system performance.

## 3 Conclusion

We developed a traceless photoinitiating system that leverages 625 nm red light and an FDA‐approved dye, methylene blue, in concert with biocompatible triethanolamine. This approach enables the rapid fabrication of colorless hydrogels under ambient conditions, avoiding the residual coloration typical of conventional visible‐ and red‐light‐induced photopolymerization approaches. This has been exemplified using biocompatible GelMA, though the system can be used with other biopolymers depending on the final hydrogel application. Thanks to the utilization of red light, the system operates even through a 5 mm thick biotissue, and, therefore, can potentially be used for transdermal photopolymerization to create either drug depots for sustained drug release or encapsulate cells for tissue engineering, such as in cartilage restoration.

Combining photocuring with extrusion‐based 3D printing allowed us to print colorless, highly stable 3D structures that enable uninhibited light transmission. Finally, a 3% GelMA bioink was used for extrusion‐based 3D bioprinting, followed by photopolymerization performed at low temperatures to preserve the viability of the NIH‐3T3 fibroblasts. Cell‐laden grids cultured for ten days showed excellent viability; moreover, the fibroblasts were able to adhere to the hydrogel material. These results confirm that the described approach can indeed be utilized in tissue engineering. We also envisage that the approach can be utilized for other 3D printing technologies, such as stereolithography, though further optimization will be necessary to achieve the best possible resolution and printing speed.

## 4 Experimental Section

### Materials and Sample Preparation

Methylene blue (MB^+^), Gelatine methacryloyl (GelMA, gel strength 300 g bloom, degree of substitution 60%), and *N,N*‐Dimethylacrylamide (DMA, 99%, contains 500 ppm monomethyl ether hydroquinone as inhibitor) were purchased from Merck Darmstadt, Germany. The inhibitor was then removed by passing through a column of aluminum oxide. All other chemicals were obtained from standard suppliers and used without further purification. Triethanolamine (TEA, 99+%) was purchased from Thermo Scientific Chemicals. To prepare different TEA concentrations in the millimolar (mm) range, it was diluted in deionized water. For the preparation of 4 w/v %, 5 w/v %, 10 w/v %, 15 w/v %, and 20 w/v % GelMA, 40, 50, 100, 150, and 200 mg of GelMA were dissolved separately in 1 mL of deionized water or buffer at 40–50 °C and continuously stirred until the solution became homogeneous.

### Rheological Characterization

All rheology measurements were performed using a rotational rheometer (Discovery HR‐2, TA Instruments Inc., USA) in a parallel plate geometry with a diameter of 12 mm. For in situ photorheology, a Thorlabs LED (625 nm, M625L2) equipped with a collimator head was placed at 7 cm from the parallel plate for irradiating the sample during the measurement. Then, for measuring the storage modulus (G′) and storage loss (G″), frequency and strain were kept constant at 1 Hz and 0.1%, respectively. To measure viscoelasticity, the frequency was altered from 1 to 100 Hz. For all measurements, the same amount of sample (300 µL) was placed on the parallel plate.

In addition, the average mesh size (*ξ*, nm) and cross‐linking density (*n*_e_, mol m^−3^) were determined from oscillatory measurement results for structural analysis.^[^ ^57^, ^58^ ^]^ At 30 min after exposure to red light (44 mW cm^−^^2^), the average mesh size (*ξ*, nm) was calculated using the storage moduli (G′) of the resulting hydrogels. Equation (8) estimates the average mesh size of hydrogel at different exposure times:

(8) $$\xi ={\left(\frac{G^{\prime }N}{RT}\right)}^{-1/3}$$

where G′ is the storage modulus of the hydrogel, *N* is the Avogadro constant (6.023 × 10^23^ mol^−1^), *R* is the molar gas constant (8.314 J K^−1^ mol^−1^), and *T* is the temperature (298 K). Furthermore, the storage modulus of the linear region of the frequency sweep test was used to calculate the cross‐linking density (*n*_e_, mol m^−3^) of the hydrogels:^[^ ^51^ ^]^

(9) $$n_{e}=\frac{G_{e}}{RT}$$

where *G*_e_ is the average value of storage modulus from the linear region of oscillatory frequency sweep measurement, *R* is the molar gas constant (8.314 J K^−1^ mol^−1^), and T is the temperature (298 K). Equation (9) provides the total number of elastically active junction points within the network of the hydrogels per unit volume.

### Absorption Spectra

To follow the changes in absorption spectra during the bleaching of MB^+^, 60 spectra were measured, every 100 ms up to a total time of 6 s. A custom‐built setup was assembled, featuring a 4‐wavelength LED head (Thorlabs, LED4D006) operating as the excitation source. Additionally, a cuvette holder with a temperature control breadboard (CVH100/M, PTC1/M) and a fiber adapter (SMA905) were used. The spectra were measured with an optical fiber connected to an Avantes USB spectrometer (AvaSpec).

### 3D Printing

Prior to 3D printing, biomaterial ink formulations were filled into a 10 mL cartridge (Optimum Syringe Barrel, Nordson EFD, USA) and placed in an incubator at 37 °C for 30 min to eliminate any air bubbles. Subsequently, the cartridge was fitted into a multi‐material 3D bioprinting platform (Brinter One, Brinter AM Technologies Oy, Finland) and capped with a 200 µm plastic tapered nozzle (Nordson EFD, USA). The print head temperature was kept constant at 22 or 17 °C for GelMA 10% w/v and GelMA 4% w/v, respectively. The filament formation was observed by extruding the inks in the air with a pressure of 400–600 mbars while simultaneously recording a video of the extruded filament with the integrated Brinter camera. The 3D constructs were printed with a speed of 6 mm s^−1^ by a layer‐by‐layer deposition with in situ red light curing, using a customized Pneuma Tool Cooled print head, followed by 10 min post‐curing (at 625 nm, 44 mW cm^−2^) to stabilize the structures. The 6‐layered grid structures were imaged using an optical microscope (Zeiss Axioscope 5) with an integrated live camera (Axocam 105 color) and its image processing software (ZEN Microscopy Software) to evaluate the printability of the ink formulations. The cylinders with 17 layers (*OD* = 20 mm, *ID* = 19.6 mm, *h* = 4.2 mm), miniaturized ear (*W* = 15.99 mm, *L* = 32 mm, *H* = 6.87 mm) and staircase models (*W *= 6.25 mm, *L* = 18.84 mm, *H *= 9.38 mm) were photographed, and their dimensions were measured using ImageJ to evaluate the shape fidelity of 3D constructs.

### In Situ Nuclear Magnetic Resonance (NMR) Measurements

NMR spectra were measured on a JEOL JNM‐ECZ500R 500 MHz spectrometer (JEOL, Japan) equipped with a broadband Royal probe. The WILMAD 5 mm high‐throughput tubes were used for measurements. The number of scans was set to 16, the relaxation delay to 5 s, and the pulse angle to 45°. Measurements were performed in D_2_O (Merck). After the first measurement was complete, the sample was irradiated for a certain period and its NMR spectrum was measured again immediately after the light exposure. The phase correction and baseline correction were applied, and the chemical shifts were referenced to the residual solvent peak at 4.65 ppm. The integrals were normalized against the intensity of the residual water peak at 4.65  ppm for all samples. The signals of methacrylate at 6.55, 5.95, and 5.6 ppm were integrated, their values were summed up and compared between samples (Figures S19–S22, Supporting Information).

### Cell Culture

The NIH‐3T3 mouse fibroblast cell line (CRL‐1658, ATCC) was maintained in Dulbecco's Modified Eagle Medium (DMEM, high glucose, GlutaMAX Supplement, pyruvate; Gibco, Thermo Fisher Scientific Inc., Waltham, USA) supplemented with 10% bovine calf serum (iron supplemented, US origin, Hy‐Clone), 1 IU mL^−1^ penicillin, and 1 µg mL^−1^ streptomycin (Gibco). The cells were kept under standard conditions (37 °C, 5% CO_2_) and passaged when reaching 80% confluence using TrypLE Express Enzyme (Gibco).

### Photopolymerization Cytotoxicity Studies

Complete DMEM supplemented with 0.25 µg mL^−1^ amphotericin B and 10 µg mL^−1^ gentamicin (Gibco), DMEM‐A/G, was used as a solvent to prepare cell‐laden hydrogels containing 4% w/v GelMA, 16 µm MB, 37 mm TEA, 1–4% v/v DMA, and 2 × 10^6^ NIH­3T3 cells mL^−1^. The bioink was either incubated on ice for 30 min or processed immediately. In both scenarios, 50 µL of the bioink was pipetted onto a 3 cm Petri dish, forming a drop and cured with the red light for 10–15 min at room temperature or 4 °C. For short‐term studies, these crosslinked drops were directly analysed with live/dead imaging to determine the cell viability. In the case of long‐term studies, the drops were washed with 3 mL DMEM‐A/G for 10 min at 4 °C. Then, the medium was exchanged under aseptic conditions using a laminar flow hood, and the samples were placed in the incubator (37 °C, 5% CO_2_). A fresh medium was provided every 2–3 days, and the viability of the samples was measured over time up to day 7.

### Live/Dead Imaging

Fluorescein diacetate (8 µg mL^−1^; FDA, BLD Pharmatech Ltd., Shanghai, China) and 20 µg mL^−1^ propidium iodide (PI, BLD Pharmatech Ltd.) were dissolved in DMEM medium with 2% bovine calf serum. The cell‐laden hydrogels were immersed in the FDA/PI staining solution for 5 minutes at room temperature in the dark, followed by two washes using DMEM with 2% serum. The live/dead cells were imaged with a fluorescence microscope (Axio Zoom.V16, ZEISS, Germany). Z‐stack images were analyzed with the ZEISS ZEN 3.8 software. Cell viability was calculated based on the live/dead images according to a published protocol for automatic quantification of live and dead cells using Fiji‐ImageJ.^[^ ^59^ ^]^

### 3D Bioprinting

Bioinks containing 3 or 4% w/v GelMA, 16 µm MB, 37 mm TEA, 2% v/v DMA (all dissolved in DMEM‐A/G), and 2 × 10^6^ NIH­3T3 cells mL^−1^ were loaded into the 10cc syringe barrels (Optimum, Nordson EFD), which were then put on ice for 15 min. The syringe was equipped with a 25G tapered dispensing tip (Nordson EFD) and fitted into the Pneuma Tool Cooled print head (Brinter One) kept at 4 or 17 °C for GelMA 3% w/v and GelMA 4% w/v, respectively. 5‐layer grids (1.5 cm × 1.5 cm) were printed onto 3 cm pre‐cooled glass Petri dishes placed on a frozen metallic bed. The printing speed was 6 mm s^−1^ in all bioprinting experiments, while the bioinks were extruded with a pressure of 600–700 mbars. The printed samples were cured with the red light inside a 4 °C incubator, followed by washing with 3 mL of DMEM‐A/G for 10 min at 4 °C (two times). The cell viability was measured using live/dead imaging either directly after the printing (day 0) or over time (days 1–10). In the case of cell viability measured over time, two bioink syringes were prepared for printing. For the first syringe, cells were harvested and immediately mixed into the ink. For the second syringe, cells were placed on ice for 3 h before being mixed into ink and printed. This time delay between printing with two syringes happened due to the sample processing: each grid required ≈5 min for printing and 10 min for photocuring. The cells in the second syringe experienced more stress (longer storage outside ideal cell culture conditions), and a higher drop was observed in viability compared to the first syringe (Figure 9D, day 1). The medium and Petri dishes of printed grids were exchanged every 2–3 days under sterile conditions. Although the 3D bioprinting was performed under unsterile conditions, amphotericin B and gentamicin continuously added to the cell culture medium, efficiently prevented potential contamination.

## Conflict of Interest

The authors declare no conflict of interest.

## Supporting information



Supporting Information

## Data Availability

The data that support the findings of this study are available in the supplementary material of this article.
