# Supplementary material for: Traceless Photopolymerization with Non‐Pulsed Red Light Enables 3D‐Printable Cell‐Laden Hydrogels
Source: Adv Mater. 2025 May 16;37(30):2502386. doi: 10.1002/adma.202502386 (PMC12306389; doi:10.1002/adma.202502386)
Supplement: Supplementary file 1 — Supporting Information [file ADMA-37-2502386-s001.pdf]

ADVANCED MATERIALS

Supporting Information

for *Adv. Mater.*, DOI 10.1002/adma.202502386

Traceless Photopolymerization with Non-Pulsed Red Light Enables 3D-Printable Cell-Laden Hydrogels

Ali Eftekhari, Kelsey Rianne de Graaf, Ekaterina Takmakova, Hatai Jongprasitkul, Alexander Efimov, Sanna Turunen, Andrew Kerr, Minna Kellomäki, Robert Luxenhofer, Timo Laaksonen* and Nikita Durandin**

Traceless Photopolymerization with Non-pulsed Red Light Enables 3D-Printable Cell-Laden Hydrogels

Ali Eftekhari,[aψ] Kelsey R. de Graaf,[a,eψ] Ekaterina Takmakova,[b] Hatai Jongprasitkul,[c,f] Alexander Efimov,[a] Sanna Turunen,[c,d] Andrew Kerr,[b] Minna Kellomäki,[c] Robert Luxenhofer, *[b] Timo Laaksonen, *[a, e] Nikita Durandin*[a]

[a] *Tampere University, Faculty of Engineering and Natural Sciences, Tampere, Finland.*

[b] *Soft Matter Chemistry, Department of Chemistry, Faculty of Science, University of Helsinki, Helsinki, 00014 Finland.*

[c] *Biomaterials and Tissue Engineering Group, BioMediTech, Faculty of Medicine and Health Technology, Tampere University, 33720 Tampere, Finland.*

[d] *New Materials and Processes Group, Faculty of Engineering, Turku University of Applied Sciences, 20520 Turku, Finland*

[e] *University of Helsinki, Faculty of Pharmacy, Drug Research Program, Division of Pharmaceutical Biosciences, Helsinki, Finland.*

[f] *Chemistry-School of Natural Science and Environmental Sciences, Newcastle University, Newcastle-upon-Tyne, NE1 7RU, UK.*

* Corresponding authors: nikita.durandin@tuni.fi, robert.luxenhofer@helsinki.fi, timo.laaksonen@helsinki.fi

[ψ] AE and KG contributed equally

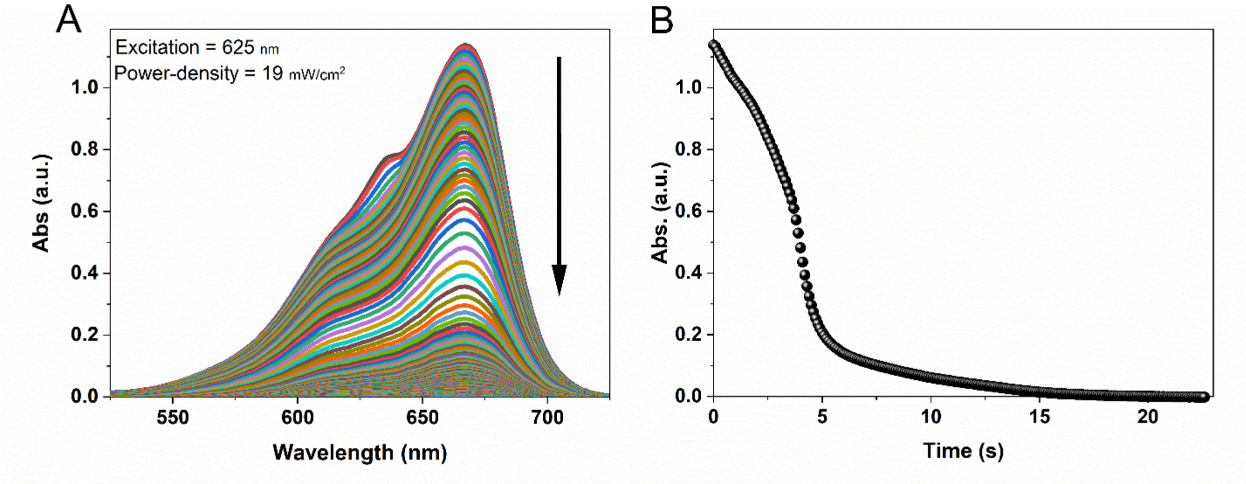

Figure S1. Real-time photobleaching of MB^+ in a solution consisting of 10% GelMA, 37 mM TEA, and 4% DMA, observed under ambient conditions. (A) Absorption spectra of MB^+ (in each 100 ms up to 6 s) measured under continuous-wave (cw) irradiation at 625 nm (19 mW/cm^2). (B) Absorbance monitored at the peaked wavelength (664 nm). Absorbance is reduced very rapidly during irradiation.

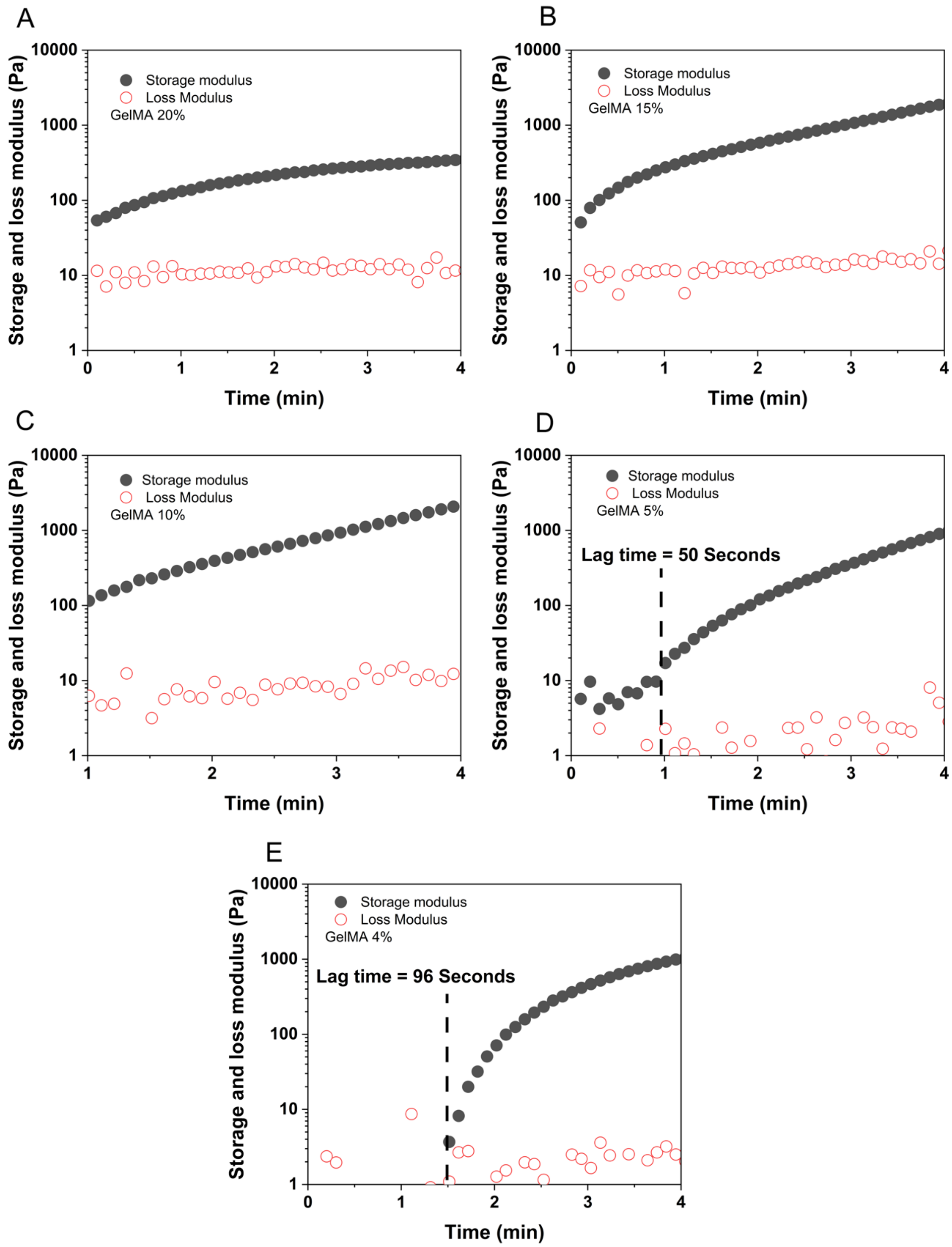

Figure S2. Effect of GelMA concentration on gelation time. There is no gelation time for GelMA concentrations of 20%, 15%, and 10% (A–C). However, for GelMA 5% and 4 %, the gelation times are 50 s and 96 s, respectively (D–E).

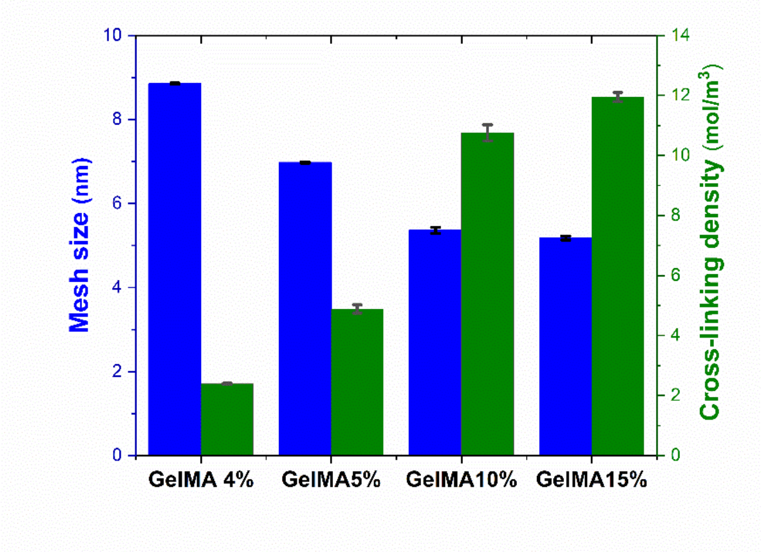

Figure S3. Calculated average mesh size and crosslinking density based on the linear region of frequency sweep measurements for different investigated GelMA concentrations. The final plateau value of the storage modulus (G′) obtained from a single in situ photorheology measurement was used to calculate the average mesh size and crosslinking density. Once G′ reached a stable plateau, ten consecutive data points (n = 10) were collected from that region to calculate the mean and standard deviation. The error bars represent the standard deviation across those ten plateau data points.

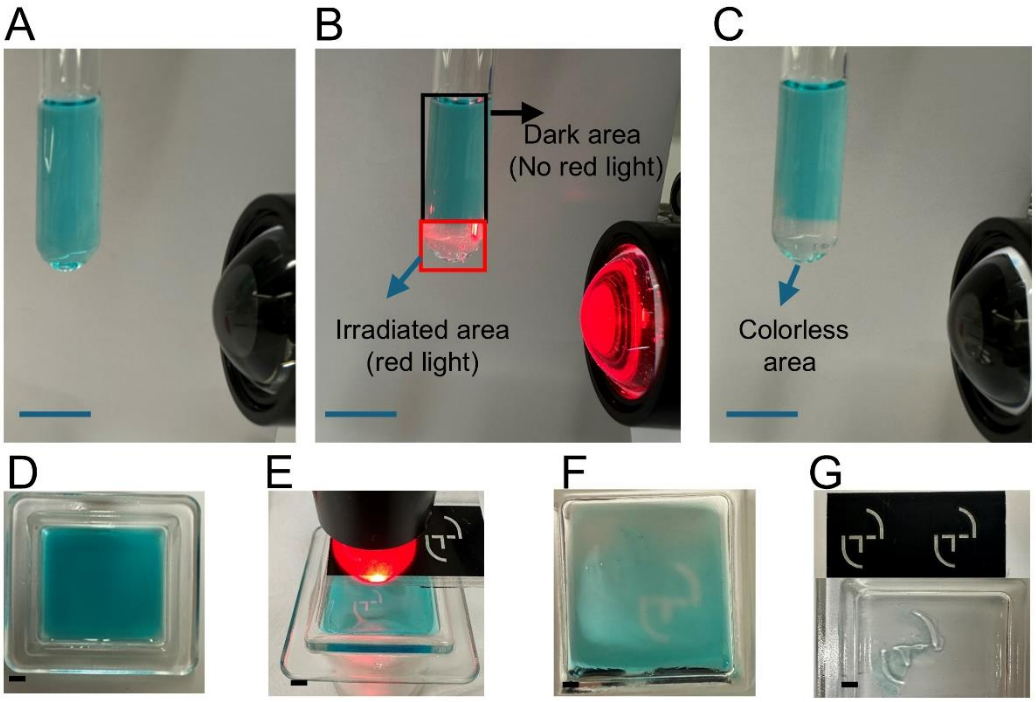

Figure S4. (A–C) A test tube filled with the ink was partially irradiated for 2 minutes using red light at 27 mW/cm², resulting in a colorless region where exposure occurred. (D–G) Using the same ink formulation, a university logo (TAU logo) served as a photomask to demonstrate spatial control; only the exposed area crosslinked and became colorless, confirming the system's effectiveness. The ink formulation contained 4% w/v GelMA, 37 mM TEA, 4% v/v DMA, and 60 µM MB^+. A higher MB^+ concentration was chosen to enhance image contrast. For the photorheology measurements, the same chicken pieces were positioned in front of the LED, 7 cm away from the sample, to ensure complete coverage of the LED head (Figure 7B). Both scale bars: 5 mm.

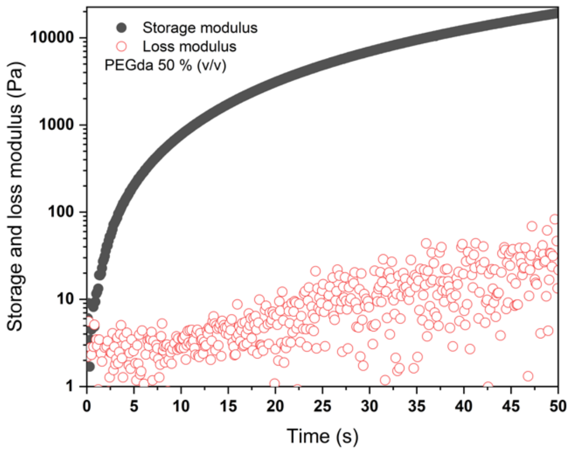

Figure S5. Photopolymerization kinetics and mechanical properties (storage modulus, G′, and loss modulus, G″) of 50 % polyethylene glycol diacrylate (PEGDA, Mn 750) using the MB^{+}/TEA photoinitiation system upon 625 nm irradiation (44 mW/cm²).

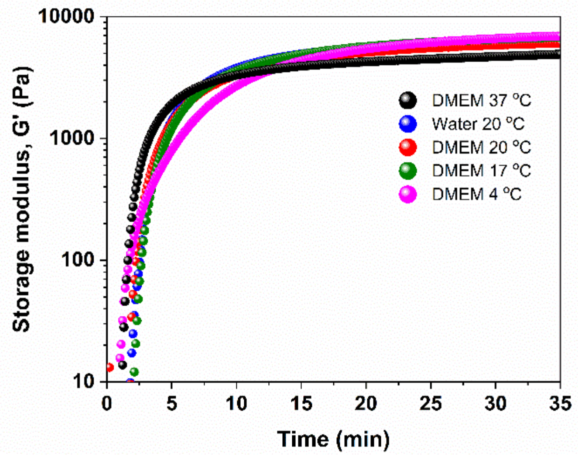

Figure S6. Investigation of reaction kinetics and mechanical properties of GelMA 4% at different media and temperatures. By changing the medium from water to DMEM, no adverse effects, such as radical scavenging and/or radical chain transfer, were observed. In addition, by decreasing the temperature to 17 °C, the photopolymerization kinetics and final storage modulus remained unchanged. This experiment demonstrates the possibility of extrusion-based 3D printing with GelMA while cooling the ink to 17 °C. The measurements were performed in 4% w/v GelMA60, 37 mM TEA, and 4% DMA solution.

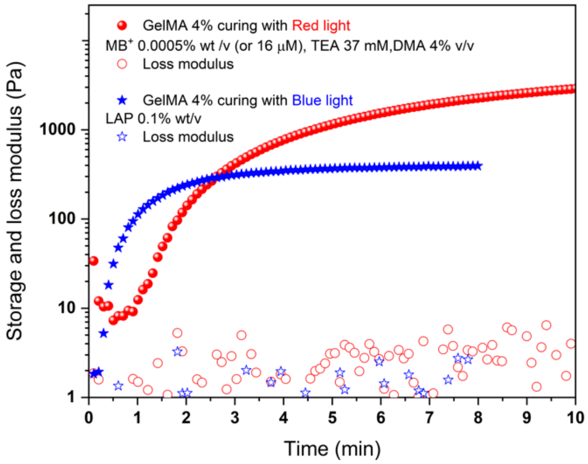

Figure S7. Comparison of photopolymerization kinetics and mechanical properties (storage modulus, G′, and loss modulus, G″) between hydrogels crosslinked with red light (625 nm, MB^+/TEA) and blue light (405 nm, LAP photoinitiator), both at a power density of ~40 mW/cm². GelMA concentration was 4% w/v in both cases.

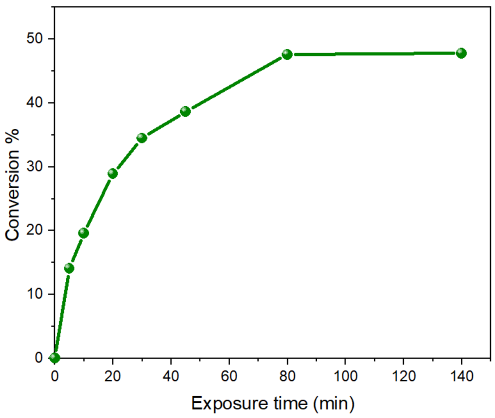

Figure S8. *In situ* ^{1}H-NMR spectroscopy measurements of monomer conversion in a photopolymerization reaction. The calculated percentage of monomer conversion over time (green). The measurements were conducted on a solution containing 10% w/v GelMA60, 16 µM MB, 15 mM TEA, and 4% v/v DMA in D_2O, with a power density of 30 mW/cm² at a wavelength of 625 nm.

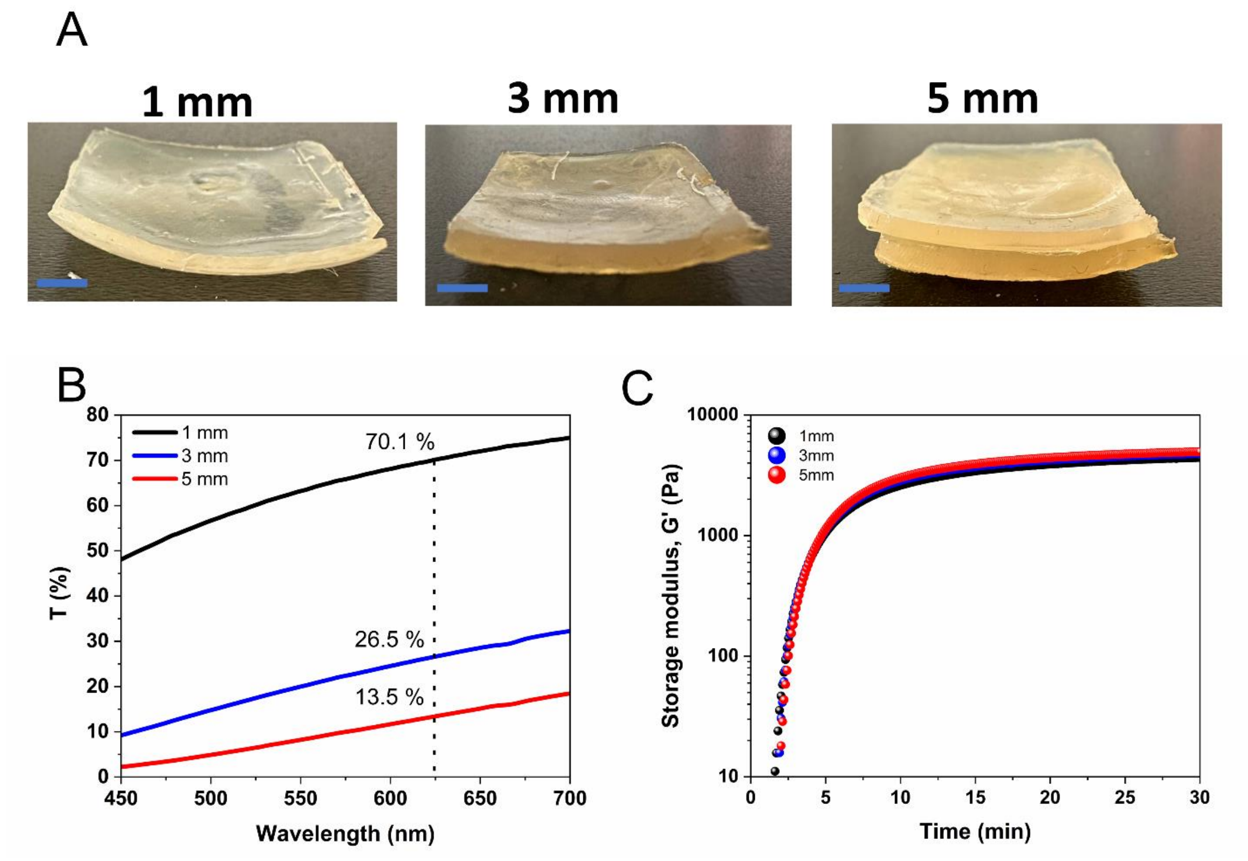

Figure S9. (A) Utilizing 5% agar gel as a tissue-mimic in three thicknesses to simulate light-tissue interactions. (B) The light transmittance (T%) spectra of agar gel pieces were measured. (C) Oscillatory time sweep measurements of 4% w/v GelMA upon red light irradiation at 625 nm through various agar gel thicknesses. All measurements were conducted in GelMA 4% w/v, 37 mM TEA, and 4% v/v DMA under an initial irradiation of 44 mW/cm² at 625 nm and 20 °C. Scale bars: 5 mm.

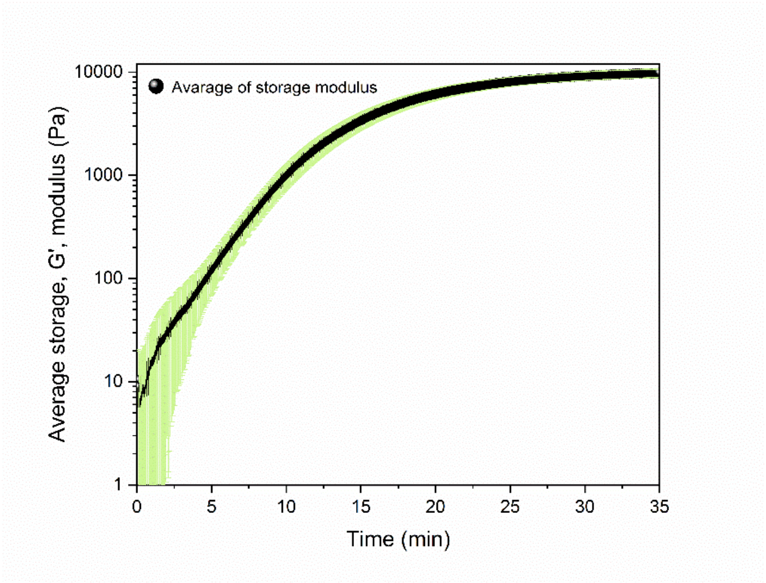

Figure S10. Photopolymerization through chicken breast with a thickness of 4.8 mm – 5 mm upon red light irradiation. Average *in situ* photorheology measurement of three pieces of the chicken breast under red light irradiation (green shade: error bars). All measurements were conducted in GelMA 10% w/v, 37 mM TEA, and 4% v/v DMA under an irradiation of 44 mW/cm^2 at 625 nm and 20 °C.

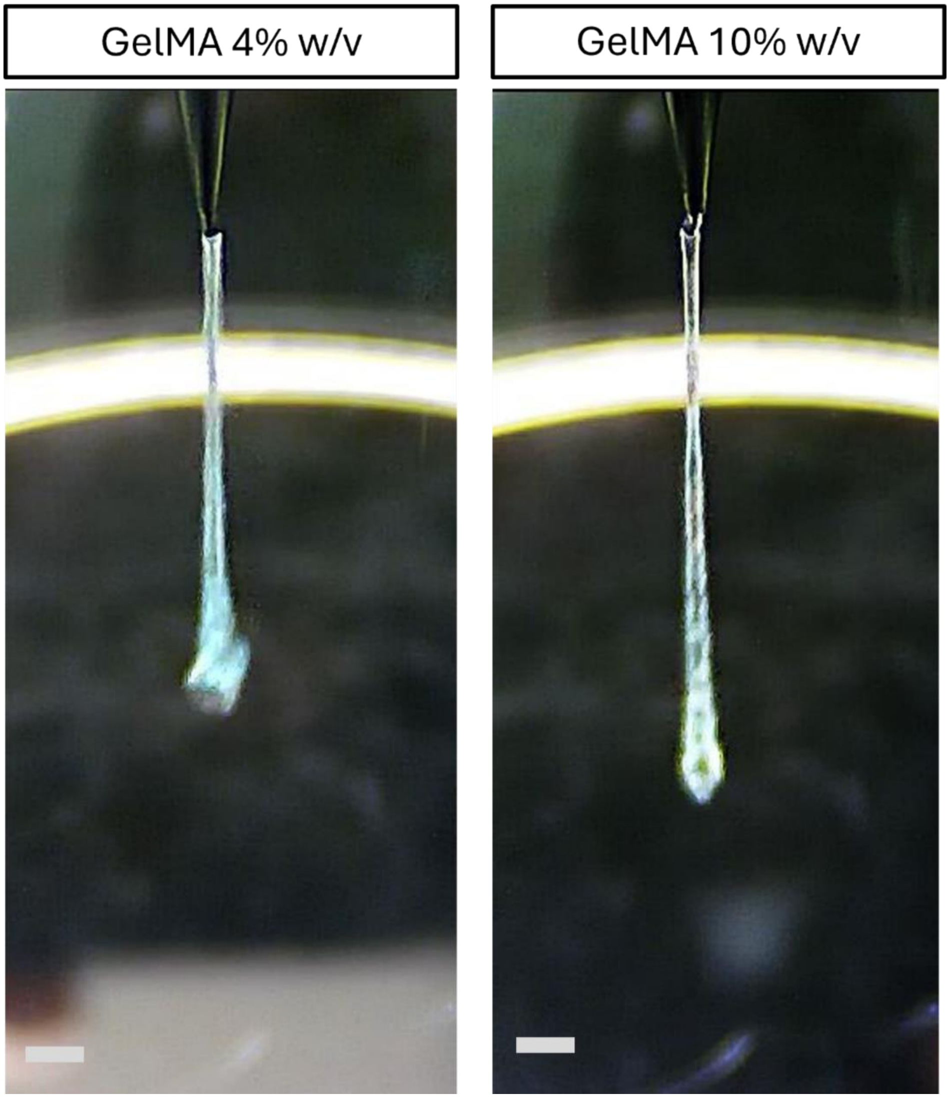

Figure S11. The filament formation of GelMA 4% w/v at 17 °C and GelMA 10% w/v at 22 °C from 200 μm tapered nozzles captured with the Brinter®-integrated online camera. Scale bars: 5 mm.

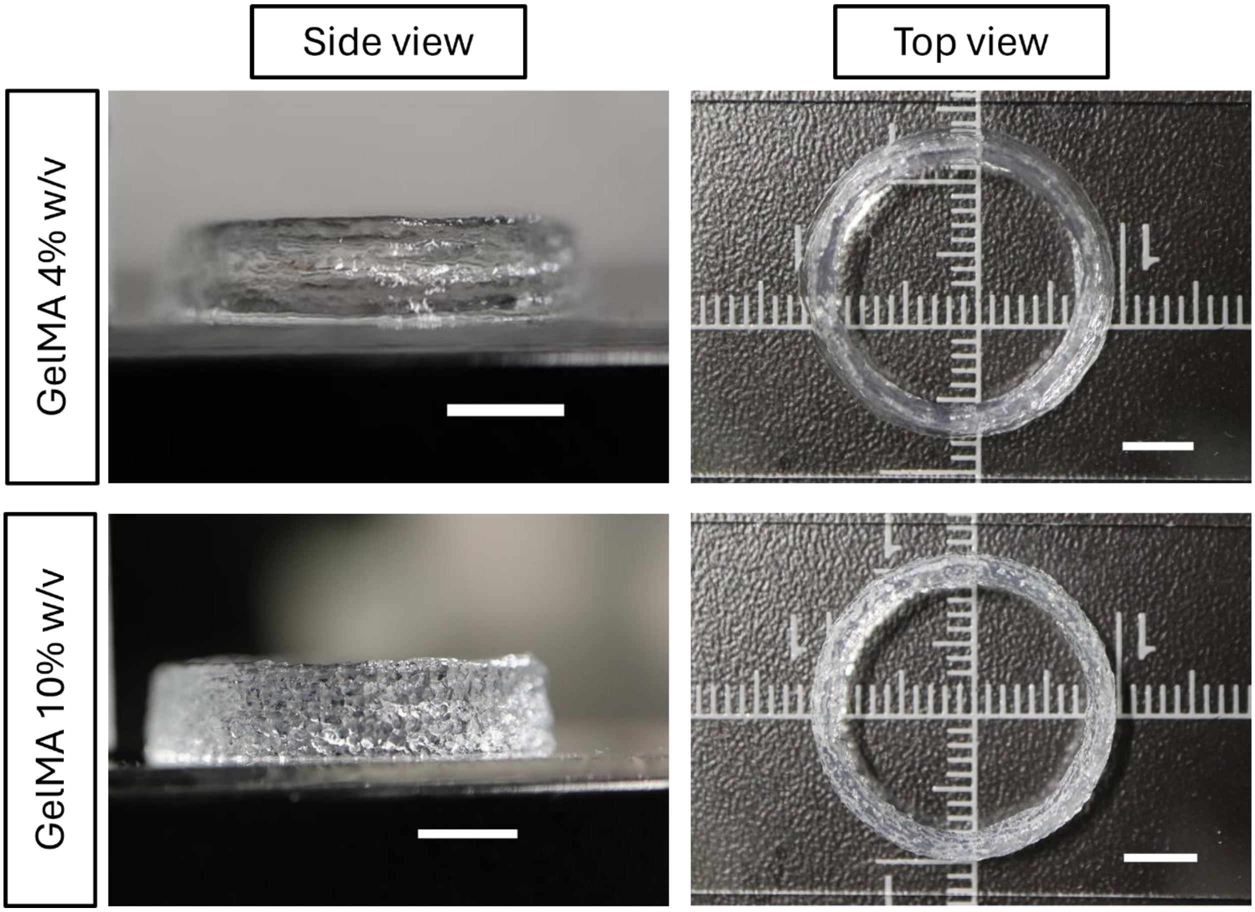

Figure S12. Side views and top views of printed GelMA 4% w/v and GelMA 10% w/v cylinders for the evaluation of shape fidelity. Ink formulation: 16 µM MB, 37 mM TEA, and 4% v/v DMA. Scale bars: 5 mm.

Table S1. Dimensions of the printed cylinders compared to the theoretical cylinder dimensions and their shape fidelity index.

CYLINDER [17 LAYERS]	THEORETICAL DIMENSIONS [MM]	4% GELMA DIMENSIONS [MM]	10% GELMA DIMENSIONS [MM]	4% GELMA SHAPE FIDELITY INDEX	10% GELMA SHAPE FIDELITY INDEX
WALL HEIGHT	4.2	4.1±0.3	4.8±0.1	0.97±0.06	1.14±0.02
OUTER DIAMETER	20.0	21.5±0.1	21.8±0.2	1.07±0.004	1.09±0.01
INNER DIAMETER	19.6	17.2±0.3	17.9±0.2	0.88±0.01	0.91±0.01

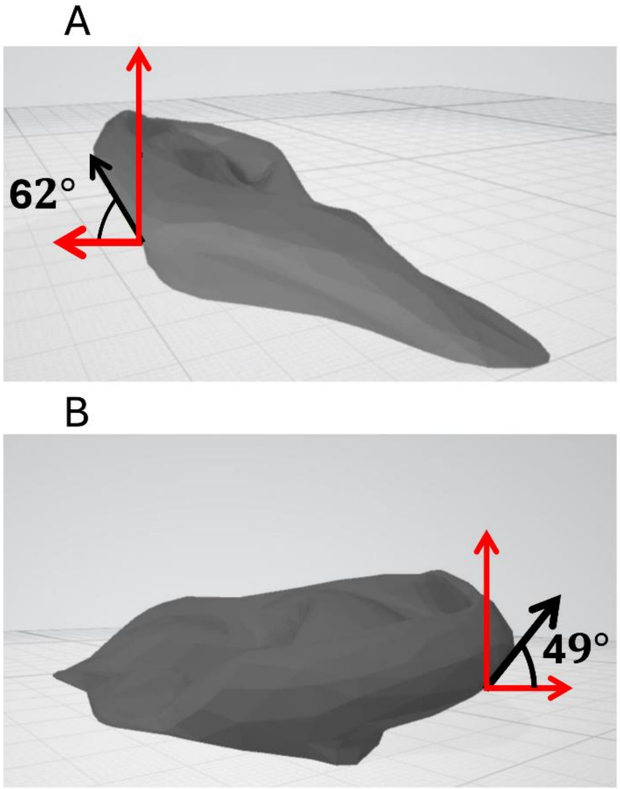

Figure S13. CAD models of two of the overhang angles of the printed ear: (A) The backside of the printed ear featured an overhang of ~62°, while the top of the ear had an overhang of ~49°.

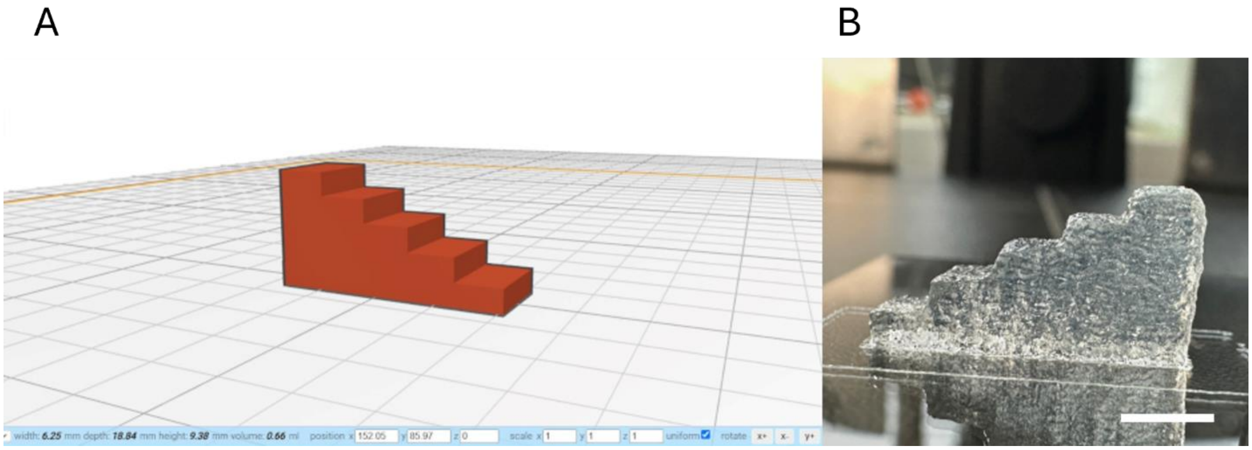

Figure S14. The CAD model and photo of the printed staircase from GelMA 10% w/v for evaluating shape fidelity of more complicated geometry. Ink formulation: 16 µM MB, 37 mM TEA, and 4% v/v DMA. Scale bar: 5 mm.

Table S2. Dimensions of the printed staircase model compared to the theoretical dimensions and their shape fidelity index.

STAIRCASE [31 LAYERS]	THEORETICAL DIMENSIONS [MM]	MEASURED DIMENSIONS [MM]	SHAPE FIDELITY INDEX
**HEIGHT**	9.38	9.30±0.37	0.97±0.01
**WIDTH**	6.25	6.69±0.27	1.07±0.04
**LENGTH**	18.84	17.40±0.39	0.92±0.02

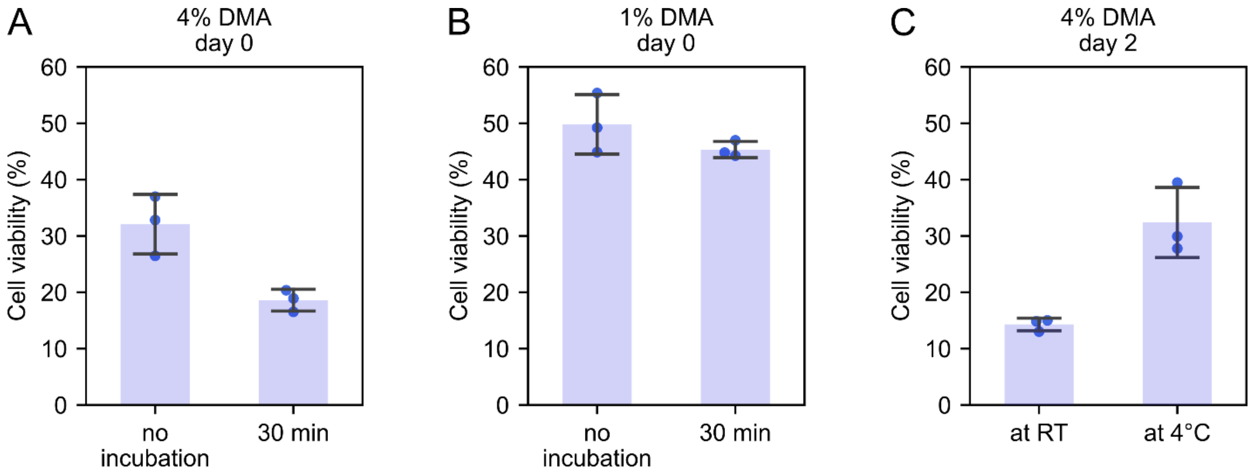

Figure S15. The viability of NIH-3T3 fibroblasts in 4% w/v GelMA hydrogel (16 µM MB, 37 mM TEA) with 1 or 4% v/v DMA. (A, B) The 4% w/v GelMA with (A) 4% v/v DMA or (B) 1% v/v DMA hydrogel was mixed with $2 \cdot 10^6$ cells/mL. The bioink was either immediately treated with red light (no incubation) or after being incubated on ice for 30 minutes (30 min). The curing was done at room temperature for 10 minutes. The cells were stained with FDA/PI. The viability was determined based on the microscope images of live and dead cells. (C) The 4% w/v GelMA with 4% v/v DMA hydrogel was mixed with $2 \cdot 10^6$ cells/mL. The bioink was immediately treated with red light at room temperature (at RT) or at low temperature (at 4 °C). The cell-laden crosslinked hydrogels were cultured for two days, and the cell viability was measured using live/dead imaging. The data is shown as mean ± SD (n = 3); each dot represents an image.

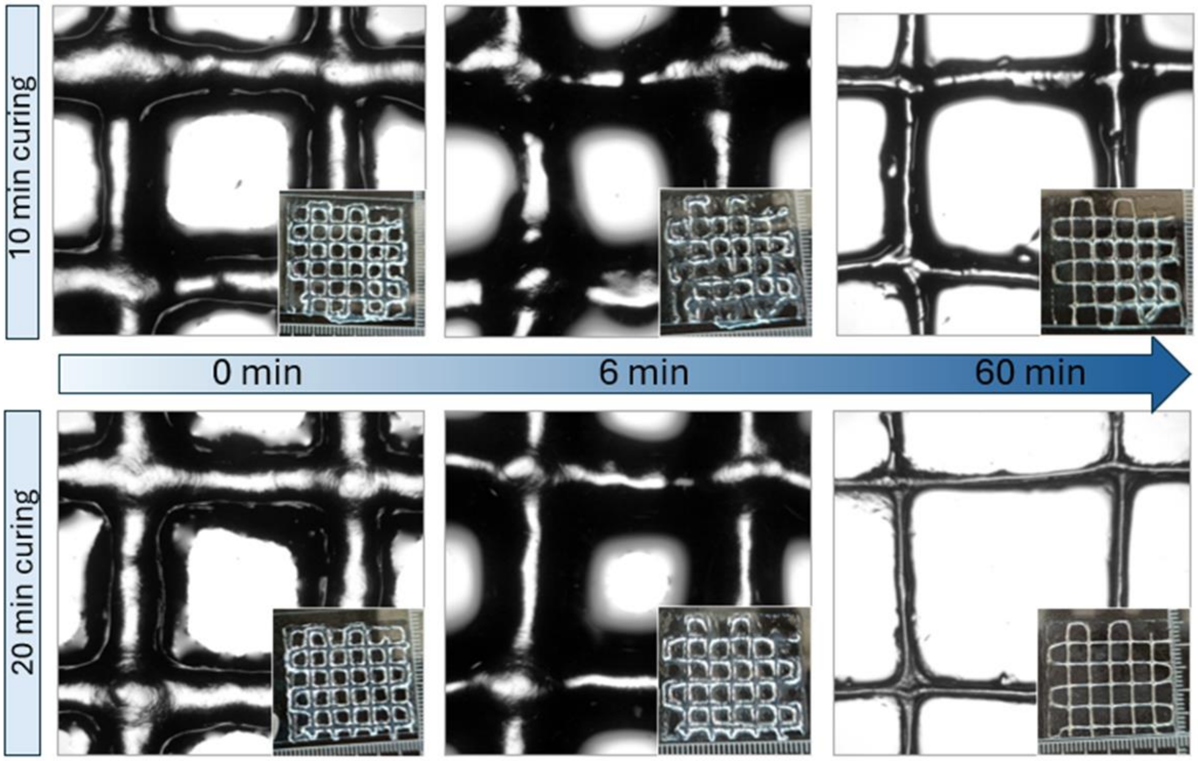

Figure S16. The printability and stability of cytocompatible printing protocol: 3% w/v GelMA, 16 μM MB, 37 mM TEA, and 2% v/v DMA grids printed with a 4 °C print head after photocuring for 10 minutes or 20 minutes with a power density of 30 mW/cm² at a wavelength of 625 nm. Stability was evaluated by drying in a 37 °C incubator.

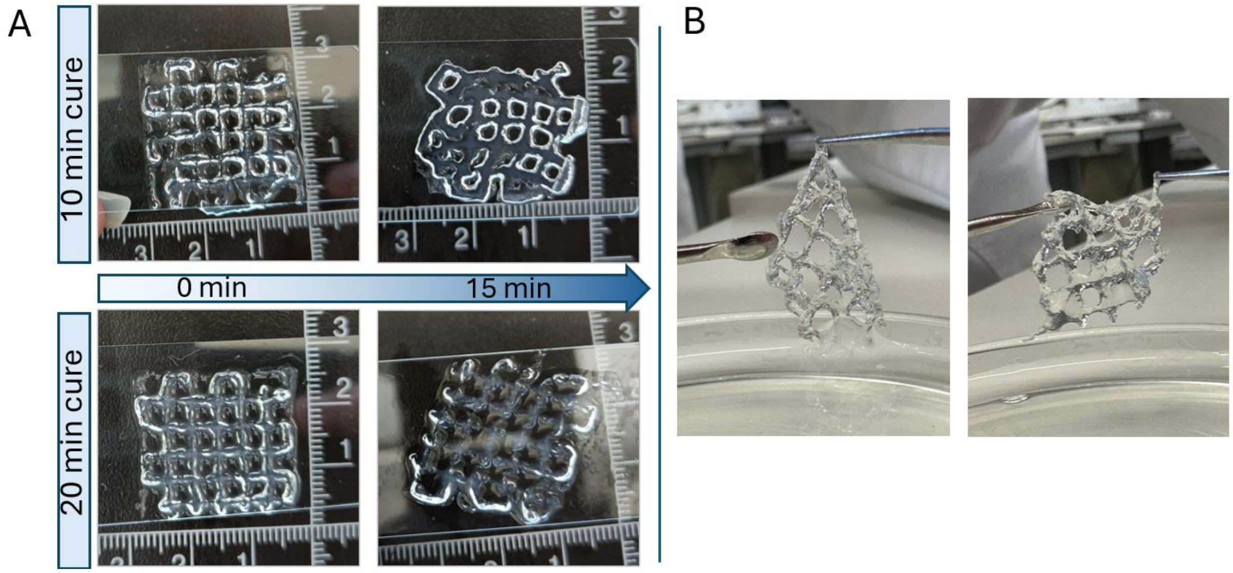

Figure S17. The printability and stability of a cytocompatible printing protocol: 3% w/v GelMA, 16 µM MB, 37 mM TEA, and 2% v/v DMA grids were printed with a 4 °C print head and photocured for 10 minutes or 20 minutes with a power density of 30 mW/cm² at a wavelength of 625 nm. (A) Stability was evaluated by soaking in a 37 °C water bath. (B) Printed GelMA grid structures were dried, rehydrated, and held with tweezers.

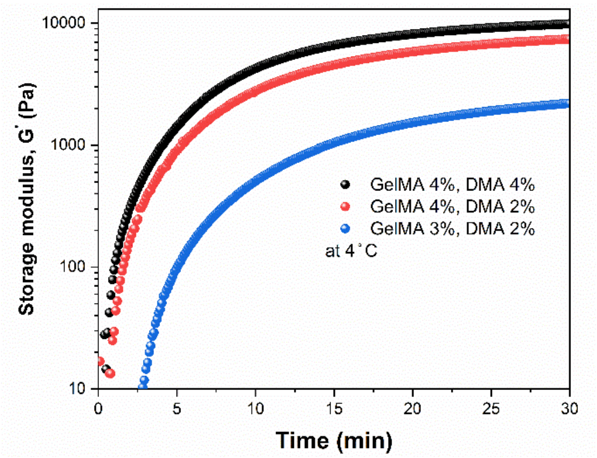

Figure S18. Investigation of the reaction kinetics and mechanical properties of 3% w/v and 4% w/v GelMA at different DMA concentrations (4% v/v and 2% v/v) at 4 °C. The results show that in 4% GelMA, reducing the DMA concentration by half does not significantly affect the reaction kinetics. Conversely, reducing the GelMA concentration to 3% slows the reaction kinetics, although it still reaches approximately 1 kPa within 15 minutes.

NMR Spectroscopy section: The polymerization reaction was monitored using ^{1}H-NMR. It should be noted that the composition used for polymerization contained three different acrylic groups (Figure 5A, main text): free dimethylacrylamide **1**; methacrylic esters **2** at hydroxyprolines and hydroxylysines of gelatine; methacrylic amides **3** at lysines and arginines of gelatine. All three types of acrylic double bonds produce the ^{1}H-NMR peaks in a narrow region 5.5–6.5 ppm, and their signals overlap. It can be found from the literature that the gelatin-methacrylamides show peaks at 5.5. and 5.7 ppm, and the gelatin-methacrylate show peaks at 6.1 and 5.7 ppm.[1] In our case, due to the reagent ratio used for polymerization, the signals of dimethylacrylamide at 6.55, 5.95, and 5.6 ppm were predominant in the spectrum by a large margin (Figure S20 and Figure S21). The only clear baseline-separated signal of the gelatine-bound methacrylic groups was a multiplet at 6.35 ppm (Figure S21). Its integral was 0.5 relative to the integral 100 of the vinyl proton of DMA **1** in the initial, non-illuminated composition.

For the quantitative evaluation of polymerization, a sample of 4% w/v GelMA, 16 µM MB, 37 mM TEA, and 4% v/v DMA solution in D2O was used. First, the NMR spectrum was measured from the non-irradiated sample. The NMR tube was then illuminated with red light (625 nm, 30 mW/cm^{2}) for 5 min, and the NMR spectrum was measured again. The sample for irradiated for another 5 minutes, 15 minutes, 25 minutes, 40 minutes, 75 and 135 minutes, and the NMR spectra were recorded between the measurements. The total illumination time was therefore 0, 5, 10, 20, 30, 45, 80 and 140 minutes. Changes of the signal intensities at 6.55, 5.95 and 5.6 ppm were monitored over the seven measurements. The integral values were normalized by the intensity of the residual deuterated water peak at 4.65 ppm as an internal standard, to which a value of 1000 was assigned for every spectrum (Figure S22). The decrease in the double bond signal intensities was clearly observable and it reflected the diminishing relative molar amount of methacrylate

double bond’s protons over time-(main text, Figure 5). Conversion % was calculated by using the equation (1):

$$\text{Conversion }\% = 100\% - I_i/I_0\times100\% \quad (1)$$

where I_0 is the integral of the signal intensities at 6.55, 5.95 and 5.6 ppm without any illumination and I_i is the integral of the signal intensities at 6.55, 5.95 and 5.6 ppm after certain illumination time.

It is noteworthy, the distinguishable signal of the gelatine-bound methacrylic groups at 6.35 ppm decreased at nearly the same rate and retained its relative ratio 1:200 to the signals of DMA. In the first 10 minutes, a rapid decrease in intensity of the double bonds’ signals and a high monomer conversion (near 20%) were observed. After 130 minutes of irradiation, the quantity of methacrylate double bonds reached a plateau**,** achieving 53% monomer conversion. Subsequently, the conversion percentage increased very slowly, if at all. This phenomenon is attributed to the high crosslinking density, which hinders the diffusion of macroradicals and thereby slows the monomer conversion process.

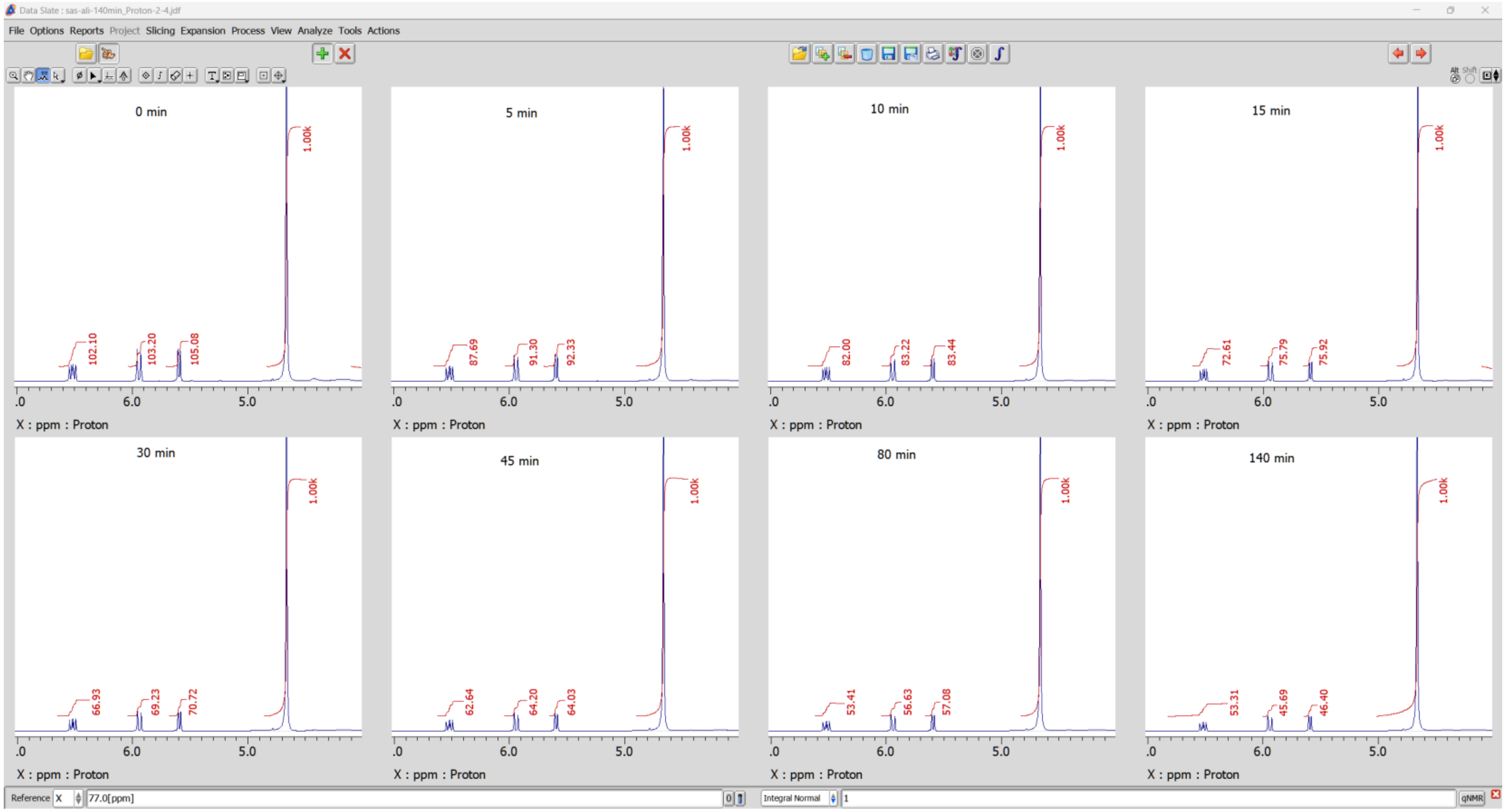

Figure S19. ^{1}H-NMR spectra measured at 0, 5, 10, 15, 30, 45, 80 and 140 minutes of irradiation with the integrals normalized to the residual solvent peak. The measurements were conducted on a solution containing 4% w/v GelMA, 16 µM MB, 37 mM TEA, and 4% v/v DMA in deuterated water, with a power density of 30 mW/cm² at a wavelength of 625 nm.

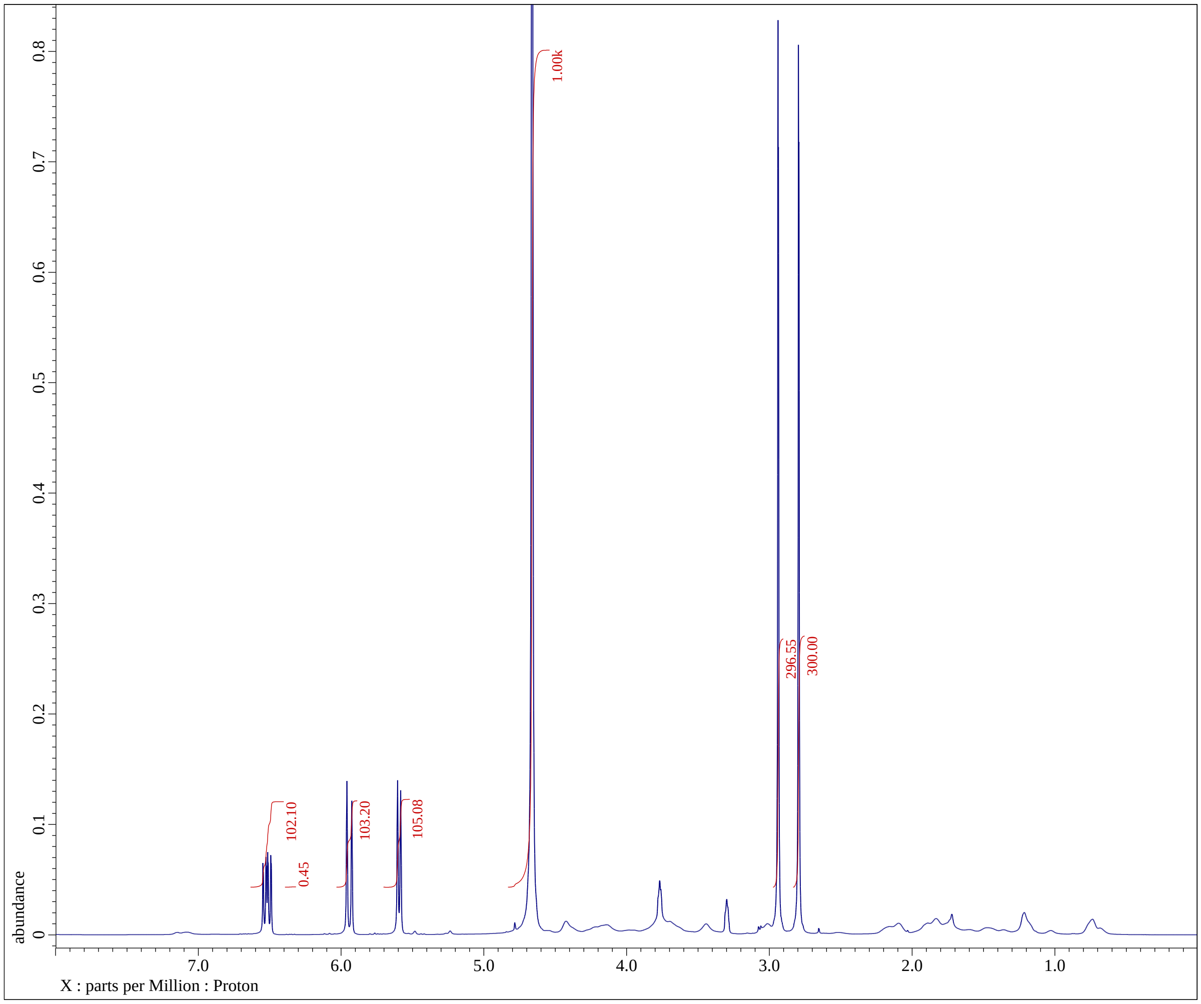

Figure S20. ^{1}H-NMR spectrum of a non-illuminated sample of 4% w/v GelMA, 16 μM MB, 37 mM TEA, and 4% v/v DMA in deuterated water.

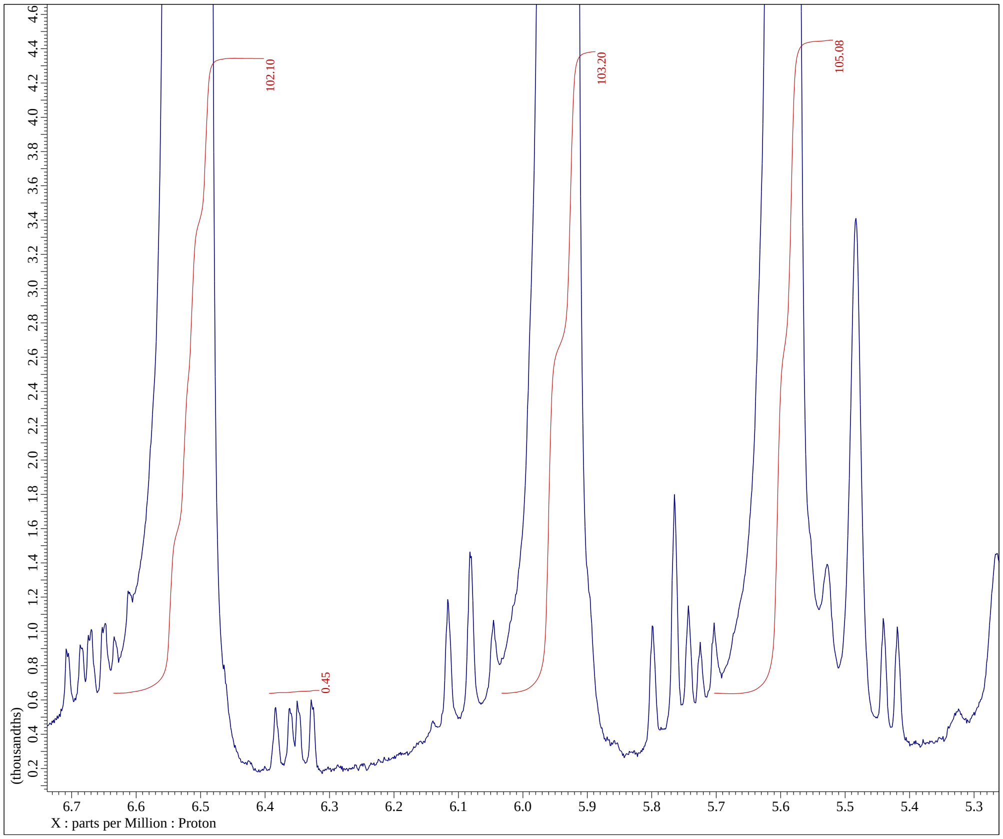

Figure S21. A zoomed-in fragment of the ^{1}H-NMR spectrum of a non-illuminated sample of 4% w/v GelMA, 16 µM MB, 37 mM TEA, and 4% v/v DMA in deuterated water, showing signals of a double bond at 6.35 ppm.

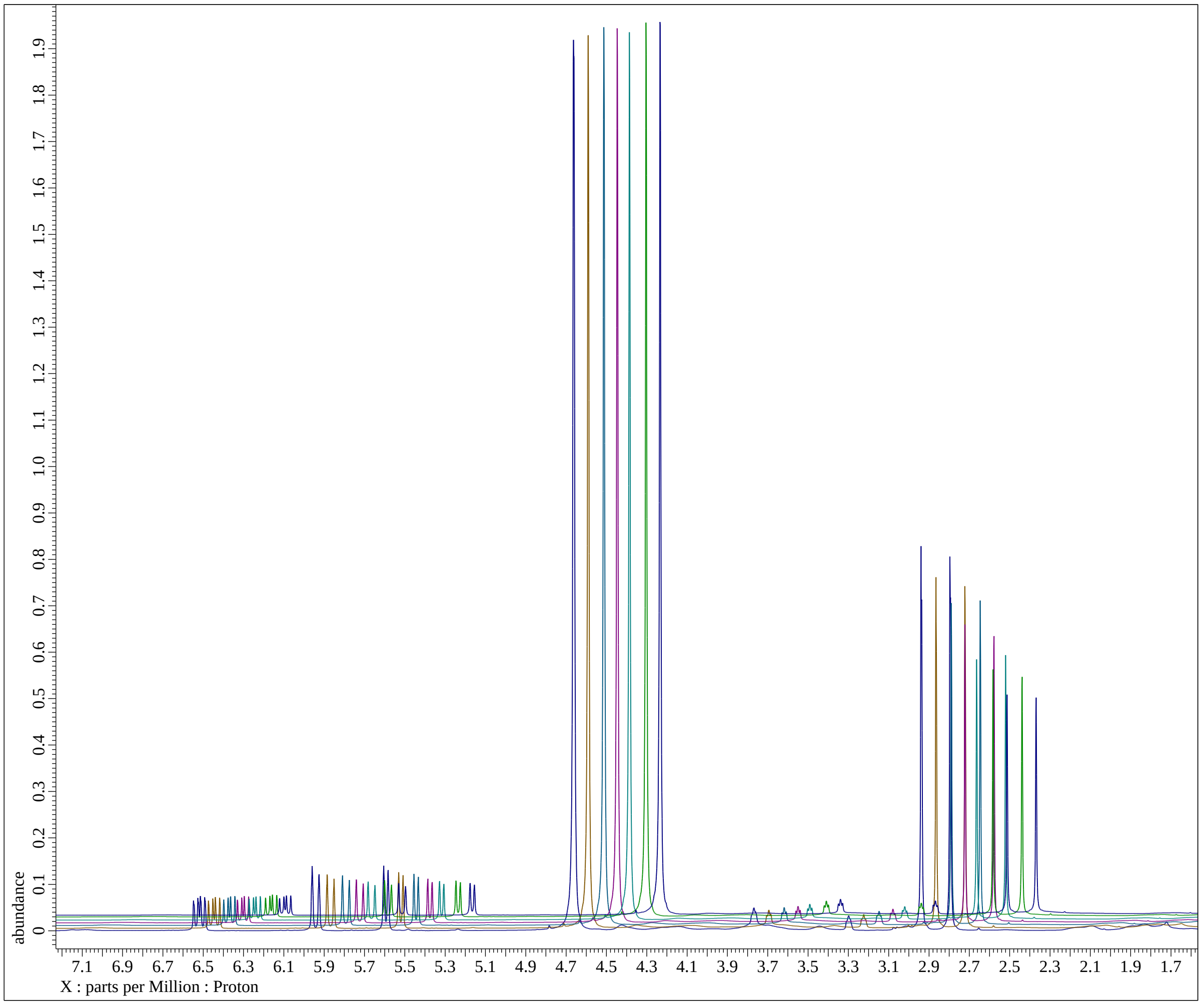

Figure S22. Gradual decrease of the ^{1}H-NMR signals of the DMA double bond with increasing illumination time. From left to right: 0 min, 5 min, 10 min, 20 min, 30 min, 45 min, 80 min.

Reference

[1] C. Claaßen, M. H. Claaßen, V. Truffault, L. Sewald, G. E. M. Tovar, K. Borchers, A. Southan, Biomacromolecules 2018, 19, 42.
